# A replacement strategy for regulating local environment of single-atom Co-S_x_N_4−x_ catalysts to facilitate CO_2_ electroreduction

**DOI:** 10.1038/s41467-023-44652-7

**Published:** 2024-01-10

**Authors:** Jiajing Pei, Huishan Shang, Junjie Mao, Zhe Chen, Rui Sui, Xuejiang Zhang, Danni Zhou, Yu Wang, Fang Zhang, Wei Zhu, Tao Wang, Wenxing Chen, Zhongbin Zhuang

**Affiliations:** 1 State Key Lab of Organic-Inorganic Composites and Beijing Advanced Innovation Center for Soft Matter Science and Engineering, Beijing University of Chemical Technology, Beijing, 100029 China; 2 Energy & Catalysis Center, School of Materials Science and Engineering, Beijing Institute of Technology, Beijing, 100081 China; 3 College of Chemistry and Materials Science, Anhui Normal University, Wuhu, 241002 China; 4 Center of Artificial Photosynthesis for Solar Fuels, School of Science, Westlake University, Hangzhou, 310024 China; 5 Shanghai Synchrotron Radiation Facilities, Shanghai Institute of Applied Physics, Chinese Academy of Science, Shanghai, 201204 China; 6 Analysis and Testing Center, Beijing Institute of Technology, Beijing Institute of Technology, Beijing, 100081 China; 7 Beijing Key Laboratory of Energy Environmental Catalysis, Beijing University of Chemical Technology, 100029 Beijing, China

**Keywords:** Electrocatalysis, Electrocatalysis, Electrocatalysis

## Abstract

The performances of single-atom catalysts are governed by their local coordination environments. Here, a thermal replacement strategy is developed for the synthesis of single-atom catalysts with precisely controlled and adjustable local coordination environments. A series of Co-S_x_N_4−x_ (*x* = 0, 1, 2, 3) single-atom catalysts are successfully synthesized by thermally replacing coordinated N with S at elevated temperature, and a volcano relationship between coordinations and catalytic performances toward electrochemical CO_2_ reduction is observed. The Co-S_1_N_3_ catalyst has the balanced COOH*and CO* bindings, and thus locates at the apex of the volcano with the highest performance toward electrochemical CO_2_ reduction to CO, with the maximum CO Faradaic efficiency of 98 ± 1.8% and high turnover frequency of 4564 h^−1^ at an overpotential of 410 mV tested in H-cell with CO_2_-saturated 0.5 M KHCO_3_, surpassing most of the reported single-atom catalysts. This work provides a rational approach to control the local coordination environment of the single-atom catalysts, which is important for further fine-tuning the catalytic performance.

## Abstract (web summary)

The local coordination environment determines the properties of single-atom catalysts. Here, authors develop a thermal replacement method to fine tune the coordination structure of cobalt-based single-atom catalysts and study their activity toward carbon dioxide electroreduction.

## Introduction

Single-atom catalysts (SACs), as emerging catalysts with unique central metal chemical environment and high central metal utilization, have shown great potential in both thermal and electrochemical catalysis^1–5^. Particularly, the SACs show high potential in the electrochemical CO_2_ reduction reaction (CO_2_RR), which is considered an appealing way to regulate atmospheric CO_2_ concentrations^6–9^. By using the SACs, high selectivity towards CO_2_RR to CO can be achieved, beneficial from the unique isolated metal active centers^10–15^. Thus, understanding the structure-performance relationship and further designing high-performance SACs is important in the CO_2_RR catalytic processes.

To design a high-performance SAC, it needs to consider not only the central metals, but also the local coordination environment of the central metals^16–19^. For a heterogeneous catalytic process, the reactants adsorb on the surface of the catalysts and convert into products. Thus, the binding energies of the catalysts to the intermediates are critical for the catalytic process^20–23^. The binding energy is determined by the electronic structure of the active center, which is controlled by its local coordination environment^24–27^. Several examples have verified the critical roles of the local coordination environment in catalytic performance. Wu et al. demonstrated that the Co-N_2_ sites were much more efficient than Co-N_4_ sites for selective reducing CO_2_ to CO^28^. A vacancy-defect Ni-N_3_-V site was investigated by Lu et al. and it was more active than the Ni-N_4_ site for CO_2_RR^29^. By replacing the coordinate atom with other atoms, the activity of the central metals could be adjusted as well. Feng et al. reported that partial displacement of N with S atoms in coordinated TM-N_x_ can greatly improve the oxygen evolution reaction activity^30^. Wang et al. illustrated that P and N co-linked Co SAC (Co_1_-P_1_N_3_) had boosted hydrogen evolution performance than the Co-N_4_ site^31^. It demonstrated that the local coordination environment of the central metal determined the catalytic performance of the SACs. However, there is still a lack of effective synthetic methods to fine-tune the local coordination environment of SACs. Unlike the homogeneous catalysts, which have metal sites in well-defined coordination environments, the local coordination environments of the SACs are difficult to control because of the various surface sites for heterogeneous catalysts^32–34^. The SACs with a given coordination environment are always synthesized by a specific route, but it is difficult to synthesize a series of SACs with adjustable coordination environments. The SACs with asymmetry coordination environment with different linkages are particularly difficult to control their structure, due to the different reactivity of the coordinate atoms in the synthesis. More controllable synthetic methods for SACs are desired but still challenging.

Here, we demonstrate a thermal replacement strategy to fine-tune the local coordination environment of the SACs, and a series of SACs with controllable local coordination environment Co-S_x_N_4−x_ (x = 0, 1, 2, 3) were successfully synthesized. The symmetrical Co-N_4_ was generated first, and the additional S substituted N at elevated temperature to form the S substituted Co-S_x_N_4−x_ SACs. The gradual replacement process guaranteed the precise control of the local coordination environment of the SACs, and based on these series of the Co-S_x_N_4−x_ SACs, the coordination-CO_2_RR performance relationship was investigated. The fine-tuned local coordination environments of the series SACs were confirmed by the X-ray absorption spectroscopy (XAS), and the valance states of the Co-S_x_N_4−x_ series were tuned continuously. The DFT calculations show that the Bader charge can be continuously adjusted by increasing the S coordination number, thus continuously enhancing the binding to the intermediates for CO_2_RR (i.e., *COOH and *CO). A volcano-type activity trend was observed versus the coordinate number of Co-S in the SACs. The Co-S_1_N_3_ has the balanced *COOH and *CO adsorption, thus giving the highest CO_2_ reduction to CO performance, which has a maximum CO Faradaic efficiency (FE_CO_) of 98% and high turnover frequency (TOF) of 4564 h^−1^ at a low overpotential of 410 mV, surpassing most of the reported SACs. This work provides an effective strategy to precisely tune the local coordination environment of the SACs, thus offering fine-tuning of the electronic structure of the central metal sites and giving promoted catalytic performances.

## Results

### Syntheses and characterizations of the Co-S_x_N_4−x_ (x = 0, 1, 2, 3) SACs

**Fig. 1:**
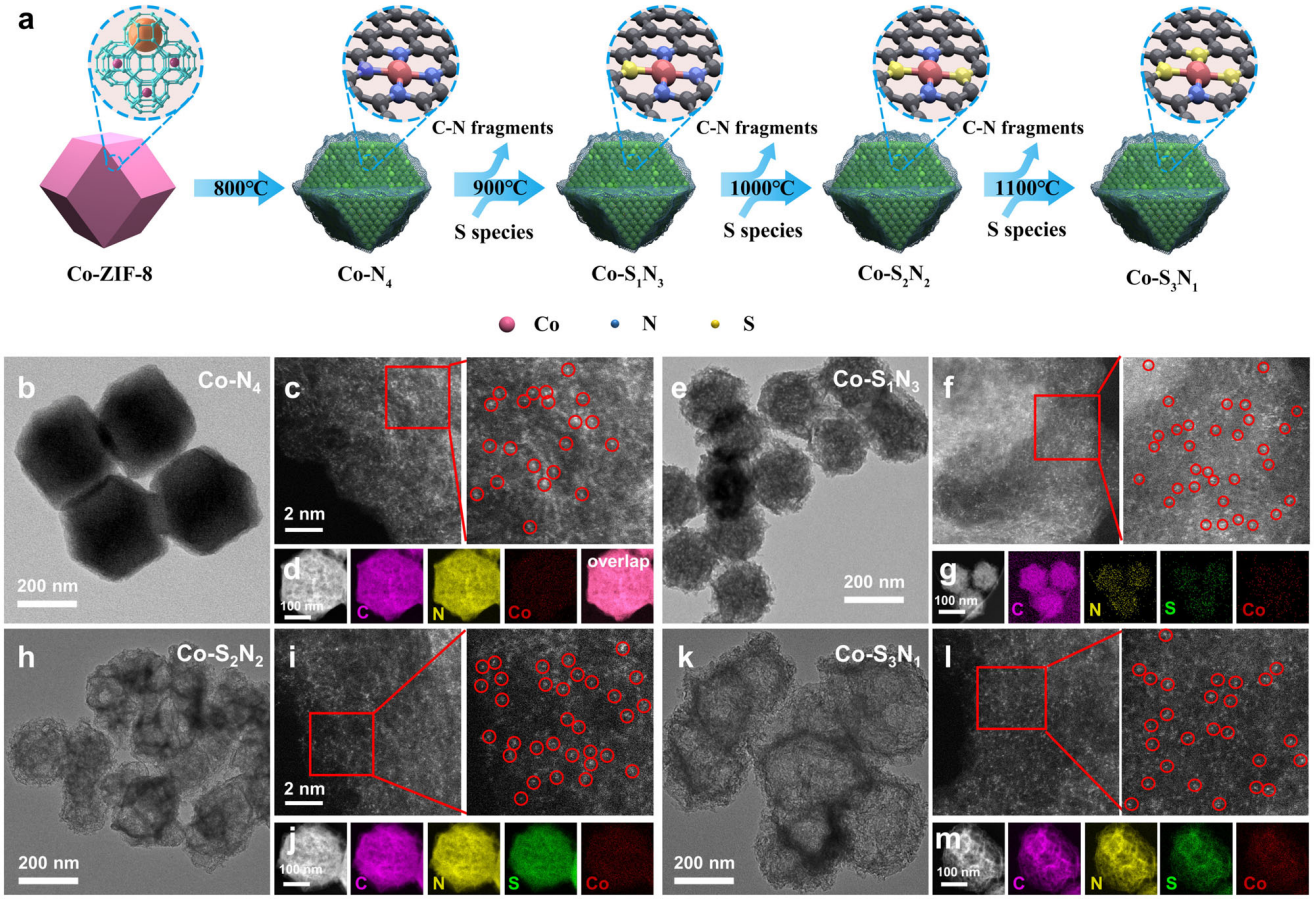
The synthesis and electromicroscopy of the Co-S_x_N_4−x_ SACs. **a** Schematic preparation procedure of Co-S_x_N_4−x_ SACs. **b**–**m** The TEM images, AC HAADF-STEM images and the EDS mappings of Co-N_4_ (**b**–**d**), Co-S_1_N_3_ (**e**–**g**), Co-S_2_N_2_ (**h**–**j**), and Co-S_3_N_1_ (**k**–**m**).

The series of SACs was synthesized by the controlled pyrolysis process of the Co-doped ZIF-8 nanoparticles. The previous studies showed that the M-N_4_ sites formed above 800 °C by pyrolysis of the metal-doped ZIF-8^35,36^. However, the loss of N dopants occurred at higher temperature^37,38^. By introducing an additional S source of thiophene, S replaced N accompanied by N lost process, thus tuning the local coordination environment of the central metal atoms. By controlling the pyrolysis temperature, the loss of N dopants was controlled, and the level of the S dopants was spontaneously controlled. As a result, a series of Co-S_x_N_4−x_ SACs with controlled Co coordinate environment, i.e., Co-N_4_, Co-S_1_N_3_, Co-S_2_N_2_, and Co-S_3_N_1_, were successfully synthesized. The synthetic approach of the series of Co-S_x_N_4−x_ SACs is schematically illustrated in Fig. 1a.

Firstly, the Co-ZIF-8 was prepared via a solution-based process, and its scanning electron microscopy (SEM) image was shown in Supplementary Fig. 1, indicating the uniform size of the products. And then, it was pyrolyzed to obtain the Co-N_x_S_4−x_ SACs under certain temperature. The Co-N_4_ SAC was obtained by direct pyrolysis of Co-ZIF-8 at 800 °C without adding S sources. The Co-S_1_N_3_, Co-S_2_N_2_ and Co-S_1_N_3_ SACs were obtained by pyrolysis with additional S sources at 900, 1000, and 1100 °C, respectively. The transmission electron microscopy (TEM) images of the obtained Co-N_x_S_4−x_ SACs are shown in Fig. 1b, e, h, and k, respectively. They show similar polyhedral morphologies with a size of ca. 200 nm. However, with the increase of the reaction temperature, i.e., with increased S contents in the products, the nanoparticles become rougher and more porous. It confirmed the broken of the Co−N moieties at high temperature, which made the materials porous^39,40^. The specific surface area and porosity were further investigated by nitrogen adsorption-desorption isotherms (Supplementary Fig. 2a), and Supplementary Table 1 listed the micropore volume, mesopore volume, Brunauer-Emmett-Teller area (*S*_BET_), micropore surface area (*S*_micro_), external surface area (*S*_ext_) for the Co-S_x_N_4−x_ SACs. The Co-S_x_N_4−x_ SACs showed comparable *S*_BET_ of 861, 919, 928, and 988 m^2^ g^−1^ for Co-N_4_, Co-S_1_N_3_, Co-S_2_N_2_, and Co-S_3_N_1_ SACs, respectively. With the increase of the S content, the *S*_BET_ slightly increases. The increase of the surface area mainly came from the increase of *S*_ext_, indicating the etching of the nanoparticles. The total pore volume increased with the increase of the S content as well, especially for the mesopores. Supplementary Fig. 2b shows the pore-size distribution curves for the Co-S_x_N_4−x_ SACs. The pores became larger with the increase of the S component, because of the etching effect at elevated temperatures.

The corresponding aberration-correction high angle annular dark-field scanning transmission electron microscopy (AC HAADF-STEM) images are shown in Fig. 1c, f, i, and l. Scattered bright dots are clearly observed, which are highlighted by red circles. These bright dots are assigned to the individual Co atoms, because of their higher atomic numbers than the C, N and S. These results suggest that Co was atomically dispersed in the carbon matrix. The corresponding energy-dispersive X-ray spectroscopy (EDS) mapping images of Co-S_x_N_4−x_ are shown in Fig. 1d, g, j and m. It reveals that Co, S, N and C were uniformly distributed in the substrates. The X-ray diffraction (XRD, Supplementary Fig. 3) patterns of the Co-S_x_N_4−x_ samples only show the peaks assigned to graphite, and no other peaks, such as attributed to CoO or CoS could be observed. It also suggests that the Co is atomically dispersed in the carbon substrates. The Co content of series Co-S_x_N_4−x_ samples was measured by ICP-OES analysis (Supplementary Table 2), presenting a similar weight percentage in the range of 0.53 to 0.56 wt%. The light element (i.e., C, H, N, S) contents of the Co-S_x_N_4−x_ SACs were measured by an elemental analyzer (Supplementary Table 3). The H contents of the Co-S_x_N_4−x_ SACs were less than 0.1%, which suggested that the precursors well carbonized during the pyrolysis processes. However, the SACs showed related high N and S contents compared with the Co contents, suggesting that the N and S were not only coordinated with Co, but also existed in the carbon substrate.

### The local coordination environments of the Co-S_x_N_4−x_ (x = 0, 1, 2, 3) SACs

**Fig. 2:**
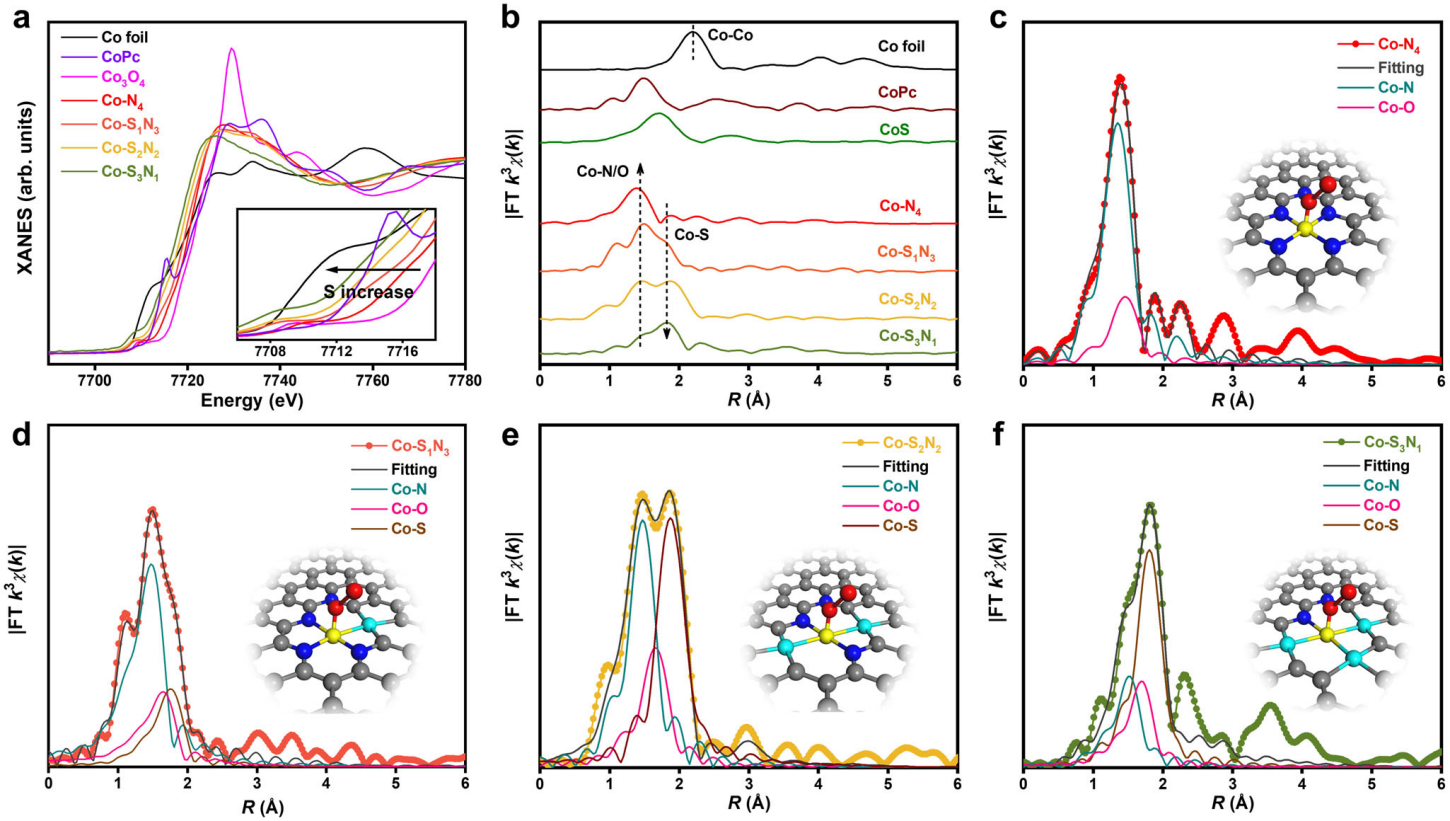
The local coordination environments of the Co-S_x_N_4−x_ SACs. **a** XANES spectra of the Co-S_x_N_4−x_ SACs. The inset is the enlarged area of the absorption-edge. **b** FT-EXAFS spectra of the Co-S_x_N_4−x_ SACs. FT-EXAFS fittings at R space of Co-N_4_ (**c**), Co-S_1_N_3_ (**d**), Co-S_2_N_2_ (**e**), and Co-S_3_N_1_ (**f**). The inset is the model for the metal sites of the corresponding SACs. Co (yellow), S (cyan), N (blue), O (red), C (grey).

The detailed local coordination environments of the obtained Co-S_x_N_4−x_ SACs were investigated by using hard X-ray absorption near-edge structure (XANES) and Fourier transform extended X-ray absorption fine structure (FT-EXAFS). Figure 2a shows the normalized Co K-edge XANES spectra of the Co-S_x_N_4−x_ and the reference samples. The inset shows the enlarged areas of the absorption edge. Compared with Co foil, the adsorption edges for the Co-S_x_N_4−x_ SACs shift towards higher energy, demonstrating the positive charges on the Co atoms. However, compared with Co_3_O_4_, they show a lower valence state of Co in the Co-S_x_N_4−x_ SACs, caused by the unique local coordination environments of the SACs. The electronic tuning effect is found on the Co-S_x_N_4−x_ with different S/N coordination numbers, demonstrating the strong sensitivity on the local coordination environments. The valence states of Co in these Co-S_x_N_4−x_ SACs decrease gradually when the amount of S increases, because of the lower electronegativity of the S than N. A sharp pre-edge peak around 7715.4 eV is observed for the CoPc with Co-N_4_ pyridinic moieties in a square planar configuration. However, this peak does not appear for the Co-S_x_N_4−x_ SACs, suggesting the adsorption of additional molecules on the Co sites in the Co-S_x_N_4−x_ SACs. Co complexes have been reported as O_2_ adsorption materials^41^. Because the synthesized Co-S_x_N_4−x_ SACs were stored in air, O_2_ may adsorb on the Co sites.

The fine local coordination environments were investigated by the FT-EXAFS (Fig. 2b). For the Co-N_4_ sample, only one peak at 1.39 Å is observed, demonstrating the single-atom structure of Co in Co-N_4_. This peak is close to the Co−N scattering (1.48 Å) observed for the CoPc. After introducing S, a peak at about 1.80 Å emerges, and its intensity increases with the S amount increase. This peak can be assigned to the Co−S scattering by the reference of CoS. Because of the larger size of S than N, the Co−S scattering is larger than that of Co−N scattering. No other peaks, such as Co−Co configuration, can be observed, confirming the Co single-atom structure of the Co-S_x_N_4−x_ series. We further extracted the structural parameters from the Co K-edge EXAFS fittings. Considering the differences of XANES and EXAFS between the Co-S_x_N_4−x_ and the standard spectra (CoPc), one O_2_ molecule was allowed to adsorb on the Co atom in perpendicular to the Co-S_x_N_4−x_ plane^42,43^. Thus, the Co−N, Co−S (from the Co coordination with the substrate) and Co−O (from the Co coordination with adsorbed O_2_) scattering paths were used in the fitting. The fitting results are shown in Fig. 2c–f and Supplementary Figs. 4-8, and the structural parameters are summarized in Supplementary Table 4. The Co−N coordination number decrease with the Co−S coordination number increase, demonstrating the replacement of N to S. The atomic structure models of Co-S_x_N_4−x_ SACs were constructed based on the fitting results and shown in the inset of Fig. 2c–f. Furthermore, we also simulated their XANES spectra that matched the experimental results well (Supplementary Fig. 9), demonstrating the successful synthesis of the series of local coordination environment fine-tuned Co-N_4_, Co-S_1_N_3_, Co-S_2_N_2_, and Co-S_3_N_1_ SACs. These results show that our thermal replacement approach can be adopted to tune the local coordination environment of the SACs.

The chemical environments of the SACs were examined by the X-ray photoelectron spectroscopy (XPS) as well. From S 2*p* XPS spectra, we found that the S atoms were successfully incorporated into the carbon substrate by this thermal replacement strategy. The S 2*p* mainly identifies the existence of Co–S and C–S–C bonds in Co-S_1_N_3_, Co-S_2_N_2_, and Co-S_3_N_1_, indicating the partial substitution of neighbouring N with S atoms (Supplementary Fig. 10a-c). The S atoms were mainly substituted for pyridinic-N, demonstrated by C and N 1 *s* XPS results (Supplementary Figs. 11, 12). Compared with Co-N_4_, the contents of pyridinic-N and Co-N_x_ in Co-S_1_N_3_, Co-S_2_N_2_, and Co-S_3_N_1_ clearly reduce with the substitution of S atoms (Supplementary Table 5). We also studied the S substitution process on the carbon substrate. When pyrolysis of graphite with the S source, no S could be detected in products from the XPS (Supplementary Fig. 13). It suggests that the S cannot be doped into the highly graphited carbon substrate. When using the nitrogen-doped carbon as the substrate, the C−S−C peaks were observed in the XPS pattern, demonstrating the S replaced the N sites in the pyrolysis processes. The Raman spectra of Co-S_x_N_4−x_ confirm the formation of disordered and graphitic carbon (Supplementary Fig. 14), which is in-line with the previously reported carbon-based SACs.

### CO_2_RR performance of the Co-S_x_N_4−x_ (x = 0, 1, 2, 3) SACs

**Fig. 3:**
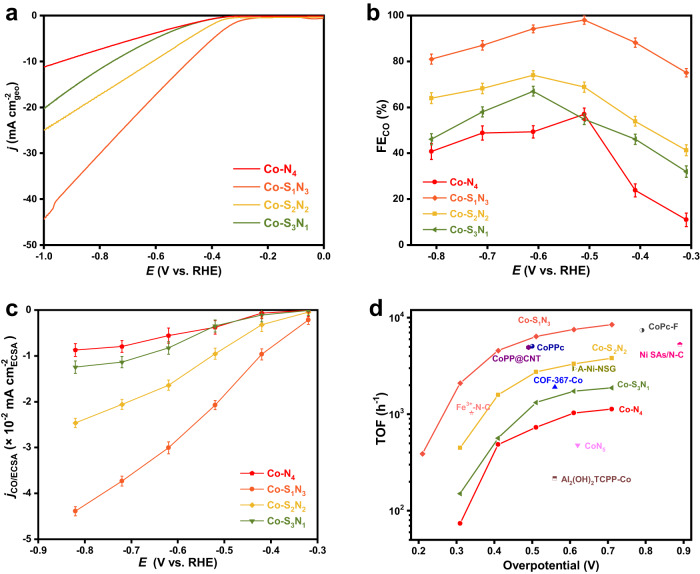
CO_2_RR performance of the Co-S_x_N_4−x_ SACs. **a** Polarization curves of the catalysts tested in CO_2_-saturated 0.5 M KHCO_3_ electrolyte. **b** The Faradaic efficiencies of CO (FE_CO_) measured by GC. **c** The corresponding the SACs, the electrochemical surface area (ECSA) normalized *j*_CO/ECSA_. **d** The turnover frequency (TOF) compared with the previously reported CO_2_RR catalysts. The reference data were obtained from refs. ^44–52^. Error bars are s.d. of at least three sets of experimental repeats.

We evaluated the CO_2_RR performance of Co-N_4_, Co-S_1_N_3_, Co−S_2_N_2_, and Co-S_3_N_1_ SACs in H-cell filled with CO_2_-saturated 0.5 M KHCO_3_ (Supplementary Fig. 15) to study the influence by the local coordination environment. Figure 3a shows the linear sweep voltammetry (LSV) curves. All potentials were *iR*- corrected, in which the solution resistance (*R*) was measured by electrochemical impedance spectroscopy and shown in Supplementary Fig. 16. A cathodic current was observed when scanned to lower potential, demonstrating the occurrence of reduction reactions. By using Co-S_x_N_4−x_ with different S and N amounts, the current density significantly changed, implying the performance is strongly correlated to the local coordination environment. The Co-S_1_N_3_ has a current density of 17.2 mA cm^–2^ at –0.6 V (vs. RHE, the same hereafter), followed by Co-S_2_N_2_ (9.6 mA cm^–2^), Co-S_3_N_1_ (4.9 mA cm^–2^), and Co-N_4_ (3.8 mA cm^–2^). The Co-S_1_N_3_ also shows the lowest onset potential of about –0.3 V relative to those counterparts. Moreover, when the catalysts were measured in Ar-saturated electrolyte, significantly reduced current density was observed, implying the cathodic current mainly from the CO_2_RR (Supplementary Fig. 17).

We further detected the gaseous products of the reaction by on-line gas chromatography (GC, Supplementary Fig. 18) and ^1^H NMR spectra (Supplementary Fig. 19), in which only gaseous products H_2_ and CO were observed rather than other liquid products. To confirm the origin of the CO, an isotopic ^13^CO_2_ feeding experiment was formed (Supplementary Fig. 20). The mass spectrum shows the peaks at 29 and 45 m/z assigned to the ^13^CO product and ^13^CO_2_ feedstock respectively, substantiating that the CO product attributed to CO_2_RR rather than side reactions such as the decomposition of the catalysts. The Faradaic efficiencies of CO (FE_CO_) were calculated based on GC results and shown in Fig. 3b. Additionally, the FE_H2_ were also calculated and displayed in Supplementary Fig. 21. The maximum FE_CO_ are 57 ± 2.6%, 98 ± 1.8%, 74 ± 2.0%, and 67 ± 2.1% for Co-N_4_, Co-S_1_N_3_, Co-S_2_N_2_ and Co-S_3_N_1_ SACs, respectively. To understand the intrinsic activity of the SACs, the electrochemical surface areas (ECSAs) of the catalysts were measured from the double layer capacitance (Supplementary Figs. 22-23. 363, 579, 471 and 451 cm^2^ for Co-N_4_, Co-S_1_N_3_, Co−S_2_N_2_, and Co−S_3_N_1_ SACs, respectively) and the ECSA normalized CO partial current densities (*j*_CO/ECSA_) were given in Fig. 3c. The different *j*_CO/ECSA_ implies the different activity of the catalysts, which were controlled by the local coordination environment of the SACs. At –0.62 V, the Co-N_1_S_3_ shows the highest *j*_CO/ECSA_ of 3.0 × 10^–2^ mA cm^–2^, which is 1.8, 3.7 and 5.4-fold to those for Co-S_2_N_2_, Co-S_3_N_1_, and Co-N_4_, respectively. The N, S-doped carbon substrates might contribute to the CO_2_RR activity as well. In order to investigate the influence of the substrates, we synthesized four control samples (CN-800, CNS-900, CNS-1000, and CNS-1100) using the similar preparation procedures for Co-S_x_N_4−x_ SACs but without adding Co sources, and then tested their CO_2_RR performance. As shown in Supplementary Fig. 24, the N, S-doped carbon substrates showed much lower CO_2_RR performances compared with their counterpart Co-S_x_N_4−x_ SACs, indicated by their lower current density, lower FE_CO, substrate_ and thus much lower *j*_CO, substrate_. It suggested that the CO_2_RR activities were mainly from the single atomic Co sites (Supplementary Table 6). And thus, the TOF of the Co-S_x_N_4−x_ SACs was calculated based on the Co sites. To eliminate the activity contribution from the substrate, we subtracted the *j*_CO, substrate_ from the total *j*_CO_, and the detailed calculation method was shown in the Method part. The TOF at an overpotential of 410 mV were 487, 4564, 1581, and 565 h^–1^ for Co-N_4_, Co-S_1_N_3_, Co-S_2_N_2_, and Co-S_3_N_1_ SACs, respectively (Fig. 3d). It is further demonstrated that the Co-S_1_N_3_ SAC exhibited the highest intrinsic activity of the Co active site for CO_2_RR, and also surpassed the most reported Co-based atomic catalysts^44–52^. In short, inspired by the continuous substitution between the neighboring N and S atoms, a series of single atom Co-S_x_N_4−x_ (x = 0, 1, 2, 3) present a volcano-type CO_2_RR activity trend, and the Co-S_1_N_3_ structure locates at the apex of the volcano with the optimal catalytic properties. In terms of the best activity and selectivity, the Co-S_1_N_3_ overcomes most of the reported CO_2_RR SACs (Supplementary Table 7).

Furthermore, the Co-S_1_N_3_ also displays excellent stability toward CO_2_RR, which still retains 95% of current density and 90% of FE_CO_ after 20 h of continuous electrolysis (Supplementary Fig. 25). After the durability test, we characterized the catalyst by HAADF-STEM, AC HAADF-STEM images, EDS mappings, XRD pattern as well as EXAFS (Supplementary Figs. 26-29). All the results show that the morphology and coordination structures of the Co-S_1_N_3_ catalyst were well maintained.

In order to evaluate the practical application of the Co-S_1_N_3_ SACs under high current density conditions, a conventional CO_2_ flow cell using gas diffusion electrodes was employed (Supplementary Fig. 30). Supplementary Fig. 31a shows the polarization curve obtained using the flow cell setup. Beneficial from the improved mass transfer, a much higher current density was achieved. For example, it reached a high current density of –200 mA cm^–2^ at −0.83 V. It showed outstanding CO selectivity with the FE_CO_ above 90% in a wide current density region from −50 to −225 mA cm^–2^ (Supplementary Fig. 31b). The Co-S_1_N_3_ SAC showed good durability in the 50 h of chronoamperometry at –0.76 V (Supplementary Fig. 31c), indicating by the slow current decay rate. The current density was –146 mA cm^–2^ at the initial and –138 mA cm^–2^ after 50 h of electrolysis, preserved at 94.8%. The FE_CO_ was measured ever 4 h of electrolysis, and maintained at a high level of 94 ± 3% throughout the 50 h of the stability test.

### The coordination-performance relationship

The above results demonstrate the significantly different CO_2_RR performance of the Co-S_x_N_4−x_ (x = 0, 1, 2, 3) SACs, implying the performance was determined by the local coordination environment of the SACs. We firstly examined that Co centers work as the active sites for CO_2_RR. The foregoing results demonstrated that the Co-S_x_N_4−x_ (x = 0, 1, 2, 3) SACs had much higher CO_2_RR performance than their N,S doped carbon counterparts, suggesting the important role of the Co sites. A poisoning experiment was also carried out by introducing SCN^–^, and a distinct depression of CO_2_RR current density was observed (Supplementary Fig. 32). No convincing poisoning effect was observed on the carbon substrate counterparts (Supplementary Fig. 33), further implying the Co centers as active sites.

We also performed in-situ XAS experiments to elucidate the local geometry of Co-S_1_N_3_ during the CO_2_RR process (Supplementary Fig. 34). The Co XANES spectra show that the near-edge absorption position shifts to lower energy and the white peak intensity decreases when the applied potentials changed from open circuit to –0.5 V and then to –0.6 V, indicating that gradual decrease of the valence state of Co is accompanied by the CO_2_RR^44,53,54^. The FT-EXAFS spectra reveal that the Co−N peak shifts to a little longer length while applying potentials to the electrode, indicating the redistribution of electrons at the Co-S_1_N_3_ atomic interface, but still maintaining the initial Co-S_1_N_3_ moiety. The extracted EXAFS fitting results are shown in Supplementary Figs. 35-37 and Supplementary Table 8. These results suggested that the Co center with a certain local coordination environment worked as the active sites for the CO_2_RR process.

**Fig. 4:**
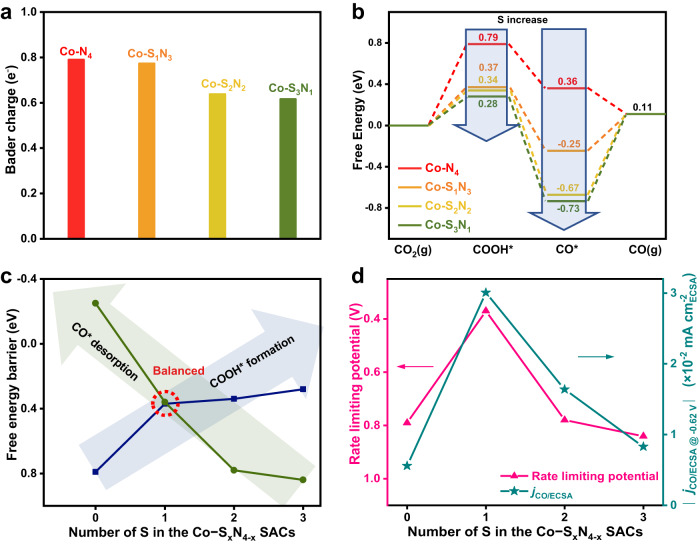
Theoretical calculated energy diagram of the Co-S_x_N_4−x_ SACs. **a** Bader charge analyses. **b** Calculated potential free energy diagrams for CO_2_RR to CO on the Co-S_x_N_4−x_ SACs. **c** Evolution of free energy of the COOH* formation and CO* desorption steps associated with the S numbers in the Co-S_x_N_4−x_ SACs. **d** Trend of the calculated limiting potentials and measured *j*_CO/ECSA_ correlated with the number of S in the Co-S_x_N_4−x_ SACs.

To understand how the local coordination environment affects the Co center, and thus affects the CO_2_RR performance of the Co-S_x_N_4−x_ SACs, DFT calculations were carried out to investigate the detailed reaction mechanism. From the ex-situ XAS measurements, an adsorbed oxygen was found on the Co sites. The Co-N-C materials were exhibited as good oxygen reduction reaction catalysts^55^. At the potential CO_2_RR occurred, the adsorbed oxygen should be reduced. Thus, we considered the Co-S_x_N_4−x_ SACs without an adsorbed oxygen at the CO_2_RR condition. The employed adsorption configuration of each species on the Co-S_x_N_4−x_ catalysts are shown in Supplementary Figs. 38–41. The charge density and Bader charge analysis were firstly examined. The charges on Co centers continuously decrease with the increase of Co−S coordination number, indicating the fine-tuning of the local coordination environment to the electronic structure of the metal centers in the SACs (Fig. 4a). These calculation results are consistent with the XANES observations.

The binding energies of the intermediates were calculated next. The CO_2_ couples with a proton and transfers an electron to form the first intermediates of COOH*, and then couples with another proton and transfer another electron to form adsorbed CO* and release a H_2_O. Finally, the CO* desorbed to generate the CO products^56,57^. The potential free energy diagrams, including the CO_2_RR key intermediates COOH* and CO*, were calculated and shown in Fig. 4b. It demonstrates that the binding energy of the COOH* and CO* intermediates strongly depends on the local coordination environment of the Co centers. With increasing the coordination number of Co-S, stronger adsorption for both COOH* and CO* was exhibited. The stronger binding energy to the intermediates promotes the adsorption steps, but hinders the desorption steps. There are mainly two endothermic steps in the reaction processes, i.e., the formation of the COOH* and the desorption of *CO. The energy barriers of these two steps are correlated to the binding energy of the COOH* and CO*, respectively. Figure 4c summarizes the energy barriers of the two steps on each SAC. With the increase of the coordination number of Co-S in the SACs, the energy barrier for the COOH* continuously decreases, and the desorption of CO* continuously increases. For Co-N_4_, it binds COOH too weakly. Thus, the formation of the COOH* is the potential-limiting step for the CO_2_RR on Co-N_4_. By substitution of N with S, the adsorption of COOH* is enhanced, thus lowering the energy barrier required for the first step. However, the adsorption of CO* strengthens accordingly, making the desorption of CO* difficult. For Co-S_2_N_2_ and Co-S_3_N_1_, the CO* desorption turns into the potential-limiting steps. Considering both factors, a volcano-shaped activity trend is observed and illustrated in Fig. 4d. The theoretical limiting potentials are 0.79, 0.37, 0.78, and 0.84 eV on Co-N_4_, Co-S_1_N_3_, Co-S_2_N_2_ and Co-S_3_N_1_ SACs, respectively. This trend is consistent with the measured CO_2_RR activity. Due to the volcano shape activity trend, it requires fine-tuning the local coordination environment of the catalysts in order to reach the apex of the volcano. With only one N substituted by S, the Co-S_1_N_3_ has a lowered energy barrier for COOH* formation and not too strong CO adsorption, showing the optimized reaction pathway to give the highest CO_2_RR performance.

Although the major trend of the calculation results to the experimental results matched well, we note there is a slight inconsistency. Based on the calculation, the Co-N_4_ should have slightly higher activity than Co-S_2_N_2_ and Co-S_3_N_1_, but the experimental results are opposed. The previous results demonstrated that the porosity of the Co-S_x_N_4−x_ SACs increases with the increase of the S content. The porous structure and large BET area ensure effective mass transfer, which is beneficial for the CO_2_RR process. Co-N_4_ has lower porosity than Co-S_2_N_2_ and Co-S_3_N_1_, which declined its CO_2_RR performance. It demonstrated that other than the major influence of the local coordination environment, the porosity influences the CO_2_RR performance of the SACs as well.

The binding to H, which is important for the competitive HER, is also adjusted by the local coordination environment of the SACs. With increasing the S coordination number, the stronger adsorption of *H was exhibited, similar to the circumstances for the CO_2_RR intermediates. The limiting potential differences between CO_2_RR and HER (i.e., Δ*U* = *U*_L_(CO_2_) − *U*_L_(H_2_), where *U*_L_ = − Δ*G*_0_/e) determine the selectivity of a catalyst in CO_2_RR^58,59^, and the values for the series of Co-S_x_N_4−x_ SACs are shown in Supplementary Fig. 42. Apparently, the Co-S_1_N_3_ catalyst has the most positive Δ*U* value, indicating the highest selectivity for CO production.

In conclusion, we developed a thermal replacement strategy to synthesize SACs with precisely controlled central Co metal local coordination environments. Systematically tuned SACs, i.e., Co-N_4_, Co-S_1_N_3_, Co-S_2_N_2_ and Co-S_3_N_1_, were successfully synthesized, and the coordination-CO_2_RR performance relationship was investigated. Beneficial from the fine-tuned local coordination environments, the binding strength to the CO_2_RR intermediates (*COOH and *CO) was continuously weakened with the increase of the coordination number of Co-S. A volcano-type activity trend was observed. The Co-S_1_N_3_ SACs located at the apex of the volcano with the optimized binding to the *COOH and *CO, and exhibited high activity and selectivity towards CO_2_RR to CO. This tailoring offers a promising approach to design and synthesize highly active electrocatalysts.

## Methods

### Chemicals

Cobalt (II) acetylacetone (Co(acac)_2_, 99%), potassium bicarbonate, Nafion D-521 dispersion (5% w/w in water and 1-propanol), 2-methylimidazole, thiophene were purchased from Alfa Aesar. Zinc nitrate hexahydrate (98%), N, N-dimethylformamide (DMF), methanol, and ethanol were obtained from Sinopharm Chemical. Nafion 117 membrane was purchased from Dupont. All the chemicals and gases were analytical grade and used without further purification. Ultrapure water (18.2 MΩ cm) was used throughout the experiment.

### Synthesis of the Co-S_x_N_4−x_ (x = 0, 1, 2, 3) single-atom catalysts

The Co-ZIF-8 was firstly synthesized. 1.07 g of Zn(NO_3_)_2_·6H_2_O and 0.12 g of Co(acac)_2_ were added into 30 ml of DMF and methanol mixture solution (volume ratio = 4:1) and stirred for 30 min. After that, 1.16 g of 2-methylimidazole dissolved in 30 ml of DMF and methanol mixture was added into the above solution and constantly stirred for 12 h. The obtained Co-ZIF-8 powders were washed with methanol several times and dried at 65 °C in a vacuum oven overnight.

The Co-ZIF-8 powder was directly annealed at 800 °C under an Ar atmosphere for 3 h to obtain Co-N_4_ SAC. For the synthesis of Co-S_1_N_3_, Co-S_2_N_2_, and Co-S_3_N_1_ SACs, 200 mg of Co-ZIF-8 powders and 0.5 ml of thiophene were added to 20 ml ethanol and sonicated for 2 h. The suspension was stirred for another 5 h at room temperature, followed by centrifuging and drying in a vacuum oven to get the S-doped precursor. Then it was pyrolyzed at 900, 1000, and 1100 °C with a heating rate of 5 °C min^−1^ under an Ar atmosphere maintained for 3 h to obtain Co-S_1_N_3_, Co-S_2_N_2_, and Co-S_3_N_1_ SACs, respectively.

### Physical characterizations

The morphology of the catalysts was monitored by transmission electron microscope (TEM, FEI Tecnai G2 F20 S-Twin) with an accelerating voltage of 200 kV as well as the field-emission scanning electron microscope (FE-SEM, JEORJSM-6700F). The HAADF-STEM images were measured by JEOL JEM-ARM200F, which worked at an accelerating voltage of 300 kV. The atomic structure of the Co-S_1_N_3_ and reference catalysts was characterized using a JEOL ARM-200CF transmission electron microscope operated at 200 keV and equipped with double spherical aberration (Cs) correctors. The crystal phases were characterized by powder X-ray diffraction (XRD, Rigaku D/max 2500Pc, Cu-K_α_ radiation, *λ* = 1.5406 Å). The X-ray photoelectron spectroscopy (XPS) measurements were performed with a Perkin Elmer Physics PHI 5300 spectrometer using Al Kα nonmonochromatic radiation. The N_2_ adsorption/desorption curves were tested at 77 K using a Micromeritics ASAP 2020 surface area analyzer. The metal content was monitored by inductively coupled plasma optical emission spectrometry (ICP-OES), which was carried out on Thermo Fisher IRIS Intrepid II. The light elements (C, H, N, and S) contents of the Co-S_x_N_4−x_ SACs were measured by an elemental analyzer (Elementar UNICUBE).

### XAFS measurements

The Co K-edge X-ray absorption measurements were conducted at the BL14W1 station within the Shanghai Synchrotron Radiation Facility (SSRF), which operates at 3.5 GeV with a maximum current of 250 mA. The X-ray radiation was monochromatized using a Si (111) double-crystal monochromator. The intensity of the incident X-ray was monitored using an N_2_-filled ion chamber (*I*_0_) positioned in front of the sample. Solid samples were placed in an aluminum sample holder sealed with Kapton tape. The data were acquired as fluorescence excitation spectra using a Lytle detector. Energy calibration was performed using the first peak maximum of the first derivative of a Co foil, which was positioned between two N_2_-filled ionization chambers (*I*_1_ and *I*_2_) after the sample. Reference spectra were recorded in transmission mode using an N_2_-filled ionization chamber. All measurements were conducted at room temperature.

In-situ electrochemical XAS measurements were conducted using a computer-controlled organic glass electrochemical cell. The working electrode utilized a catalyst-modified carbon paper, while the counter electrode was Pt wire and the reference electrode was Ag/AgCl (KCl-saturated). The carbon paper coated with Co-S_1_N_3_ was placed in contact with a copper slip, with the Co-S_1_N_3_ layer facing inward, and then the cell was filled with KHCO_3_ solution. The solutions were not agitated during the electrochemical experiment. The in-situ cell was connected to an electrochemical station using a copper tape slip protruding from the side of the working cell. A glass cap, equipped with an Ag/AgCl electrode, was used to cover the cell and maintain an appropriate distance between the working and reference electrodes for all experiments. XAFS spectra were recorded under various conditions on the electrode.

The acquired EXAFS data were processed according to the standard procedures using the ATHENA module implemented in the IFEFFIT software packages. The EXAFS spectra were obtained by subtracting the post-edge background from the overall absorption and then normalizing with respect to the edge-jump step. Subsequently, the *χ*(**k**) data of were Fourier transformed to real (*R*) space using a hanning windows (d**k** = 1.0 Å^−1^) to separate the EXAFS contributions from different coordination shells. To obtain the quantitative structural parameters around central atoms, least-squares curve parameter fitting was performed using the ARTEMIS module of IFEFFIT software packages^60–63^.

The following EXAFS equation was employed:

1 $$\chi (k)=\mathop{\sum}\limits_{j}\frac{{N}_{j}{S}_{o}^{2}{F}_{j}(k)}{k\,{R}_{j}^{2}}\exp \left[-2{k}^{2}{\sigma }_{j}^{2}\right]\exp \left[\frac{-2{R}_{j}}{\lambda (k)}\right]\sin \left[2k\,{R}_{j}+{\phi }_{j}(k)\right]$$

*S*_*0*_^*2*^ is the amplitude reduction factor, *F*_*j*_*(*k*)* is the effective curved-wave backscattering amplitude, *N*_*j*_ is the number of neighbors in the *j*^*th*^ atomic shell, *R*_*j*_ is the distance between the X-ray absorbing central atom and the atoms in the *j*^*th*^ atomic shell (backscatterer), *λ* is the mean free path in Å, ϕ _*j*_*(*k*)* is the phase shift (including the phase shift for each shell and the total central atom phase shift), *σ*_*j*_ is the Debye-Waller parameter of the *j*^*th*^ atomic shell (variation of distances around the average *R*_*j*_). The functions *F*_*j*_*(*k*)*, *λ* and ϕ _*j*_*(*k*)* were calculated with the ab initio code FEFF8.2. The coordination numbers of model samples were fixed as the nominal values. The obtained *S*_*0*_^*2*^ was fixed in the subsequent fitting. While the internal atomic distances *R*, Debye-Waller factor *σ*^*2*^, and the edge-energy shift *ΔE*_*0*_ were allowed to run freely.

The fitting parameters were listed in Supplementary Note 1.

### Electrochemical measurements for CO_2_RR

The CO_2_RR experiments were performed on a CHI 660e electrochemical workstation with an H-type cell filled with 0.5 M KHCO_3_ electrolyte. The Pt wire was used as the counter electrode and Ag/AgCl (saturated KCl) as the reference electrode. To make the working electrode, 5 mg of as-synthesized series Co-S_x_N_4−x_ SACs and 10 μL of 5% Nafion solution were introduced into a mixture containing 500 μL of water and 490 μL of isopropyl alcohol. The resulting mixture was subjected to sonication for 3 h. The working electrode was prepared by drop casting the ink on carbon paper (HCP120, 1 cm^2^), and the total loading amount of catalyst was controlled as 0.5 mg cm^−2^. Nafion 117 membrane (the membrane size was 2 × 2 cm^2^, and effective area was circular with a diameter of 1.5 cm) separated the cathodic chamber and anodic before adding electrolyte. A total volume of 30 mL of 0.5 M KHCO_3_ solution was poured into the anode and cathode chambers, and further bubbled by Ar for 30 min and then switched to CO_2_. The polarization curves were conducted at a scan rate of 10 mV s^−1^ with continuous bubbling of CO_2_ at a flow rate of 15 sccm. All the potential was reported versus the reversible hydrogen electrode (RHE) and corrected by 100% of *iR* compensation. The solution resistance (*R*) was measured by electrochemical impedance spectroscopy (EIS) across frequencies ranging from 0.1 Hz to 100 kHz. The gas products were checked via the Shimazu 2010 plus gas chromatography (GC), which was equipped with a BID detector and ShinCarbon ST 100/120 packed column. The liquid products were checked by the ^1^H NMR spectra that were collected on a Bruker AV 400 MHz NMR spectrometer using residue solvent peaks as an internal standard (^1^H NMR: D_2_O at 4.69 ppm)

A custom-built electrochemical cell with three compartments (gas, cathodic, and anodic chambers) was employed for three-electrode flow cell tests. The gas chamber thickness was 2 cm, and the anode and cathode liquid chamber have a thickness of 1 cm. A proton exchange membrane (Nafion 117) was placed between the cathodic and anodic chambers of the flow cell. The effective electrode area was 1 cm × 1 cm, resulting in an active area of 1 cm^2^. In the flow cell test, the working electrode (cathode) utilized Co-S_1_N_3_ catalyst sprayed on GDL (28 BC, Sinero Store) with a loading of 0.6 mg cm^−2^, while the counter electrode (anode) employed IrO_2_ (Sinero Store) sprayed on GDL (28 BC, Sinero Store) with a loading of 0.6 mg cm^−2^. The cathode side was supplied with electrolyte (0.5 M KHCO_3_) using a syringe pump (PHD 2000, Harvard Apparatus) at a constant flow rate of 10 sccm, while high purity CO_2_ gas was introduced at a rate of 50 sccm behind the cathode GDL. An Ag/AgCl (saturated KCl) reference electrode was utilized, and the potential was corrected by 100% of *iR* compensation.

The Faradaic efficiencies of the gas products were calculated by using the concentrations detected by the online GC as follows:

2 $${{{\mbox{FE}}}}_{{{{{\rm{CO}}}}}}\left(\%\right)=\frac{{Q}_{{{{{\rm{CO}}}}}}}{{Q}_{{{{{\rm{total}}}}}}}\times 100\%\frac{\left(\frac{\nu }{60{{\mbox{s}}}\!/\!\!\min }\right)\times \left(\frac{V}{22.4{{{{\rm{L}}}}}\!/\!{{{{\rm{mol}}}}}}\right)\times N\times {{\mbox{F}}}\times 100\%}{j}$$

where *ν* is the flow rate of CO_2_, *V* is the measured concentration of product in a 1 mL sample loop based on the calibration of the GC with a standard gas, *N* = 2 is the number of electrons, F is the Faraday’s constant (96485 C mol^−1^), *j* is the recorded current. The input flow rate was used for gaseous product FE calculations. It was assumed that the input flow rate was uniform across all currents and the cathode chamber sealed tightly.

Turnover frequency (TOF) for the CO_2_RR to CO was evaluated as follows:

3 $${{{{{\rm{TOF}}}}}}=\frac{{I}_{{{{{{\rm{CO}}}}}}}/N{{{{{\rm{F}}}}}}}{{m}_{{{{{{\rm{cat}}}}}}}\omega /{M}_{{{{{{\rm{Co}}}}}}}}\times 3600$$

where *I*_CO_ is the corrected CO partial current for the metal sites, which is subtracted the CO partial current of the N, S doped carbon substrate counterpart from the CO partial current of Co-S_x_N_4−x_ SAC, F is the Faraday’s constant (96485 C mol^−1^), *N* = 2 is the number of electrons, *m*_cat_ is the catalyst mass in the electrode, *ω* is Co loading in the catalyst, and *M*_Co_ is the atomic mass of Co (58.93 g mol^−1^).

The ECSAs was defined as the electrochemically active area of an electrode with 1 cm^2^ of geometric area. And the ECSA was calculated by the equation as follow:

4 $${{{{{\rm{ECSA}}}}}}=\frac{{C}_{{\!{{{\rm{dl}}}}}}}{{C}_{{\!{{{\rm{s}}}}}}}\times {A}_{{{{{\rm{geo}}}}}}$$

where the *C*_s_ is the specific capacitance of flat electrode, *A*_geo_ is the geometric area of the electrode (1 cm^2^). We assumed the *C*_s_ are the same for the Co-S_x_N_4-x_ and took the value of 20 μF cm^−2^ as suggested for the carbon materials based in literature^64^.

### Calculation details

The Vienna Ab Initio Simulation Package (VASP)^65,66^ was used to perform all the DFT simulations and the electron ion interaction was described with the projector augmented wave (PAW) method^67,68^. The electron exchange and correlation energy was solved by using the revised Perdew-Burke-Ernzerhof (RPBE) exchange-correlation functional within the generalized gradient approximation^69^. An energy cut-off of 400 eV and a second-order Methfessel-Paxton electron smearing with σ = 0.2 eV were used to guarantee the accuracy. The convergences criteria for energy and force are set to be 10^–5^ eV and 0.02 eV Å^−1^, respectively. A vacuum layer of 12 Å was set between the periodically repeating slabs. Spin polarization was included throughout all the calculations.

The Co-N_4_ catalyst was modeled by a *p* (4 × 4) supercell of graphene doped with nitrogen and Co atoms, where Co coordinated with four neighboring N atoms as shown in Supplementary Fig. 38. Because of the high N contents, the configurations with an additional N to replace a carbon atom in the substrate were employed. As comparisons, the Co-S_1_N_3_, Co-S_2_N_2_ and Co-S_3_N_1_ catalysts were also modeled by a *p*(4×4) supercell with the four neighbor N atoms replaces by one, two and three S atoms as shown in Supplementary Figs. 39-41. The atomic coordinates were upload as txt files as Supplementary Data files. The (3 × 3 × 1) Monkhorst–Pack grid K-points was used to sample the Brillouin zone for all four catalysts. The most stable adsorption configurations of COOH, CO and H species on the Co-N_4_, Co-S_1_N_3_, Co-S_2_N_2_ and Co-S_3_N_1_ catalysts were also given in Supplementary Figs. 38–41. The free energies of all the reaction mechanisms in this work were calculated with computational hydrogen electrode (CHE) model as developed by Norskov group^70,71^.

### Supplementary information

Atomic Coordinates

Supplementarty Information

## Data Availability

All data in the article and supplementary information are available from the corresponding authors upon request.
