# Supplementary material for: A replacement strategy for regulating local environment of single-atom Co-SxN4−x catalysts to facilitate CO2 electroreduction
Source: Nat Commun. 2024 Jan 10;15:416. doi: 10.1038/s41467-023-44652-7 (PMC10776860; doi:10.1038/s41467-023-44652-7)
Supplement: Supplementary file 2 — Supplementarty Information [file 41467_2023_44652_MOESM2_ESM.pdf]

Supplementary Information for

A Replacement Strategy for Regulating Local Environment of Single-atom Co−S_xN_{4-x} Catalysts to Facilitate CO_2 Electroreduction

Pei *et al*.

Supplementary Figures

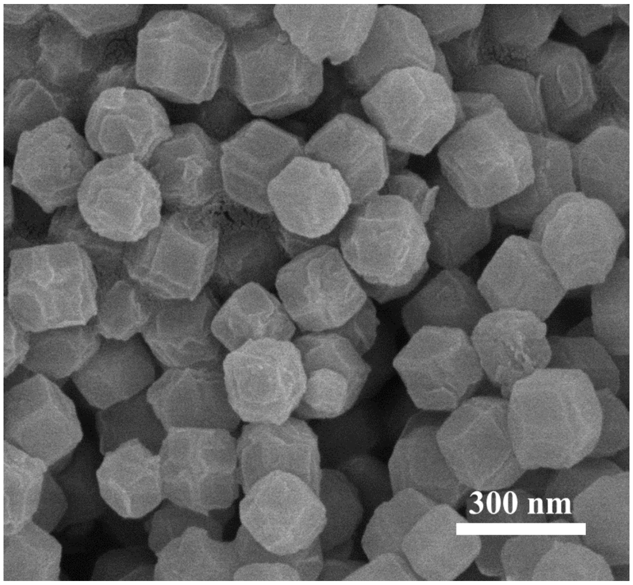

Supplementary Fig. 1. The scanning electron microscope image of Co-ZIF-8 sample.

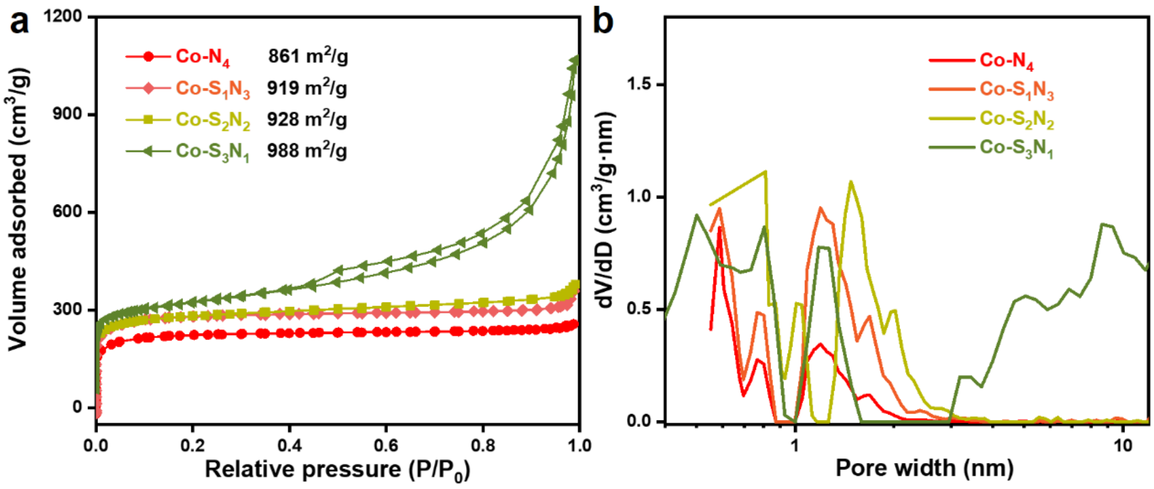

Supplementary Fig. 2. (a) The N_2 adsorption and desorption isotherms for Co−N_4, Co−S_1N_3, Co−S_2N_2, and Co−S_3N_1 SACs (b) The corresponding the pore-size distribution curves.

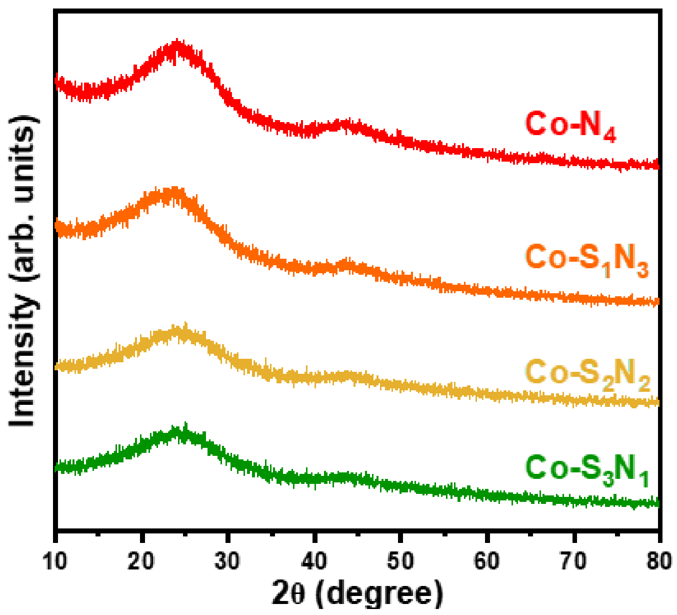

Supplementary Fig. 3. XRD patterns of Co−N_4, Co−S_1N_3, Co−S_2N_2 and Co−S_3N_1 SACs. The broad peaks at 20-30° and 40-50° are assigned to the (002) and (101) plane of graphite, respectively.

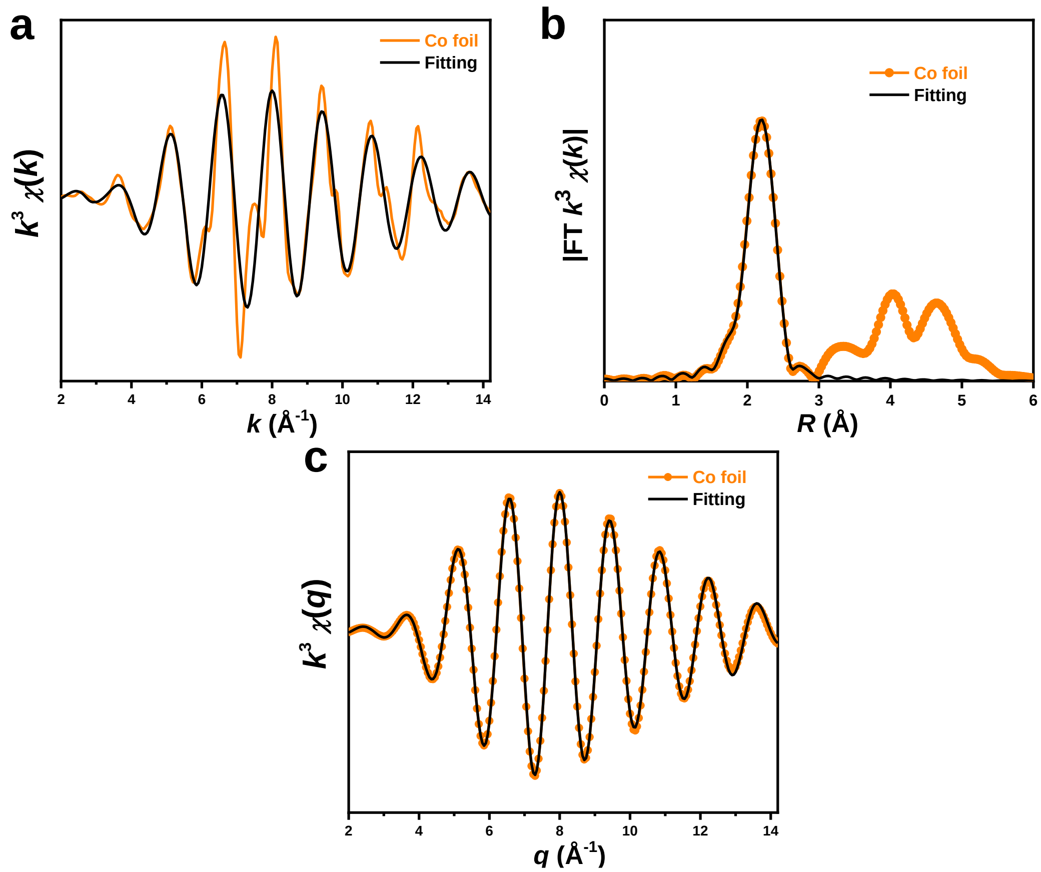

Supplementary Fig. 4. The FT-EXAFS fitting spectra of Co foil. (a) FT-EXAFS fitting at *k* space. (b) FT-EXAFS fitting at *R* space. (c) FT-EXAFS fitting at *q* space.

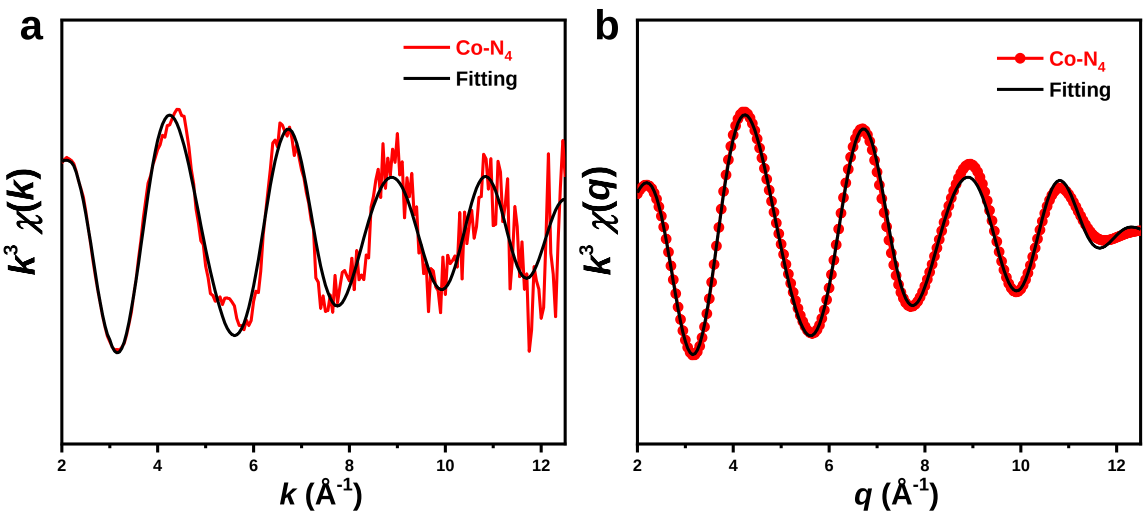

Supplementary Fig. 5. The FT-EXAFS fitting spectra of Co−N_4. (a) FT-EXAFS fitting at *k* space. (b) FT-EXAFS fitting at *q* space.

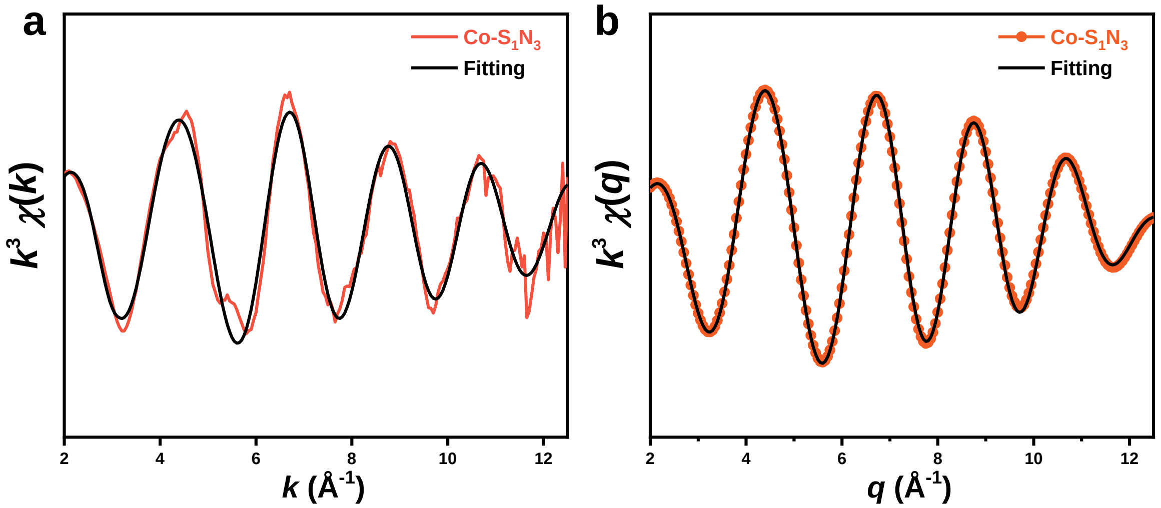

Supplementary Fig. 6. The FT-EXAFS fitting spectra of Co−S_1N_3. (a) FT-EXAFS fitting at *k* space. (b) FT-EXAFS fitting at *q* space.

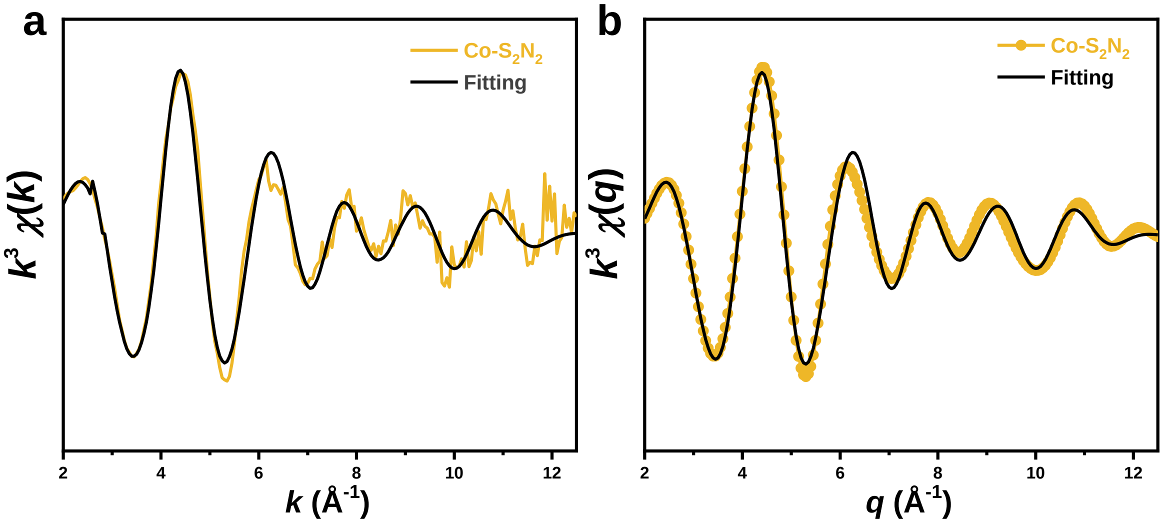

Supplementary Fig. 7. The FT-EXAFS fitting spectra of Co−S_2N_2. (a) FT-EXAFS fitting at *k* space. (b) FT-EXAFS fitting at *q* space.

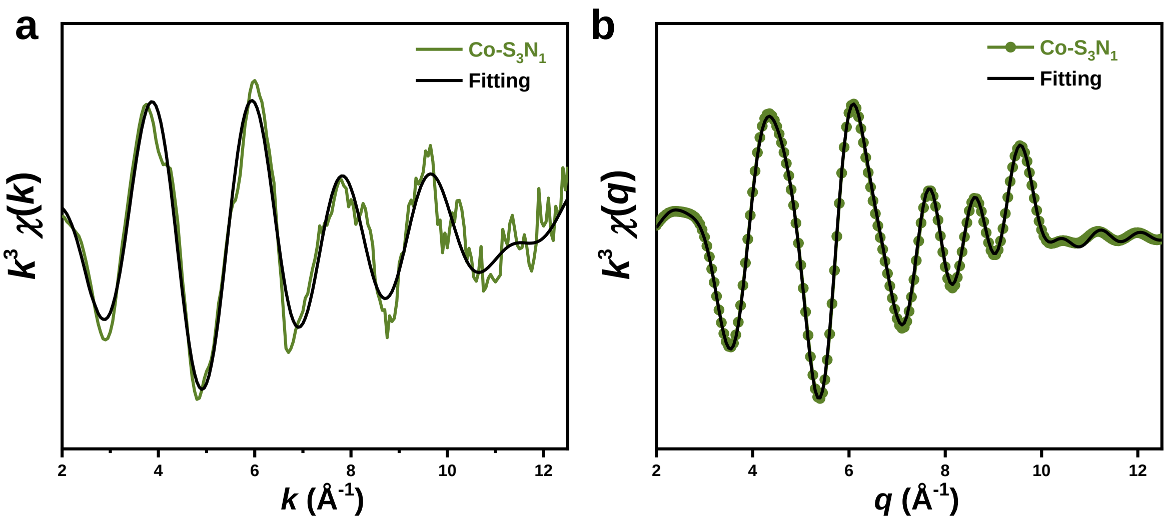

Supplementary Fig. 8. The FT-EXAFS fitting spectra of Co−S_3N_1. (a) FT-EXAFS fitting at *k* space. (b) FT-EXAFS fitting at *q* space.

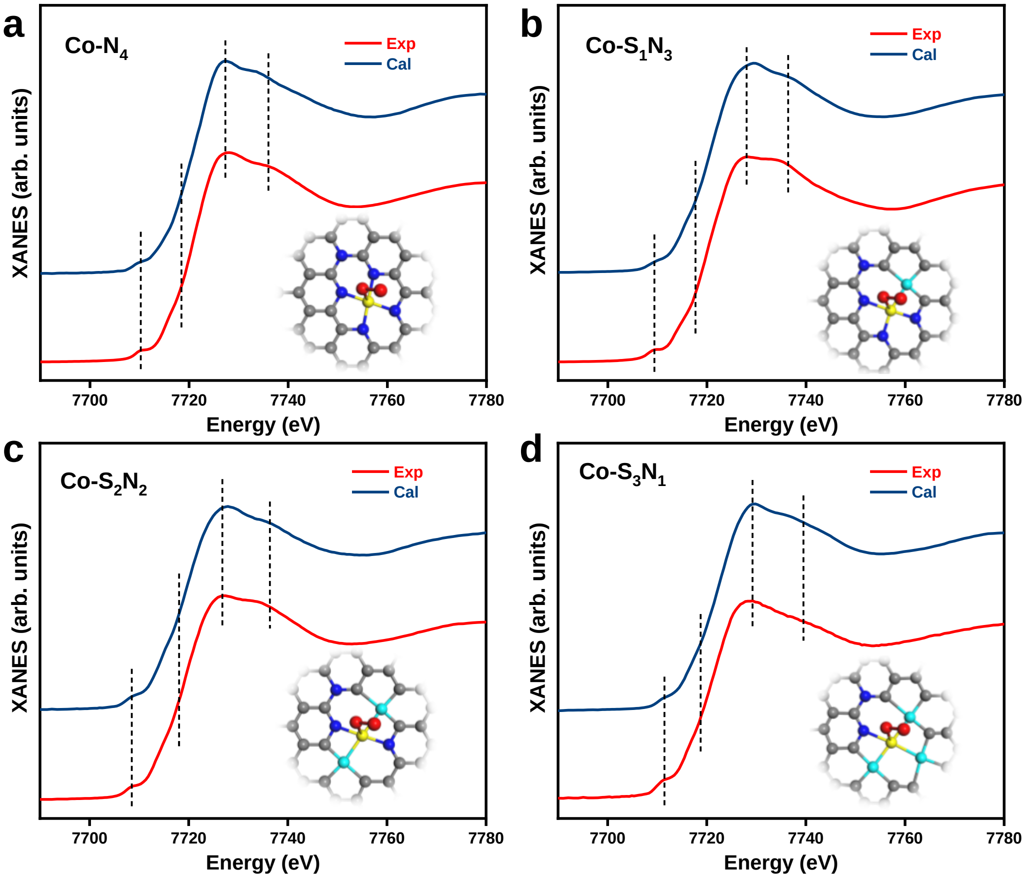

Supplementary Fig. 9. Experimental and theoretically calculated XANES curves of Co−N_4, Co−S_1N_3, Co−S_2N_2, and Co−S_3N_1 SACs at Co K-edge. The insets are the models used for calculation. The Co K-edge theoretical XANES simulations were carried out with the FDMNES code in the framework of real-space full multiple-scattering (FMS) scheme using Muffin-tin approximation for the potential.[1-3] The energy dependent exchange-correlation potential was calculated in the real Hedin-Lundqvist scheme, and then the spectra convoluted using a Lorentzian function with an energy-dependent width to account for the broadening due both to the core-hole width and to the final state width.

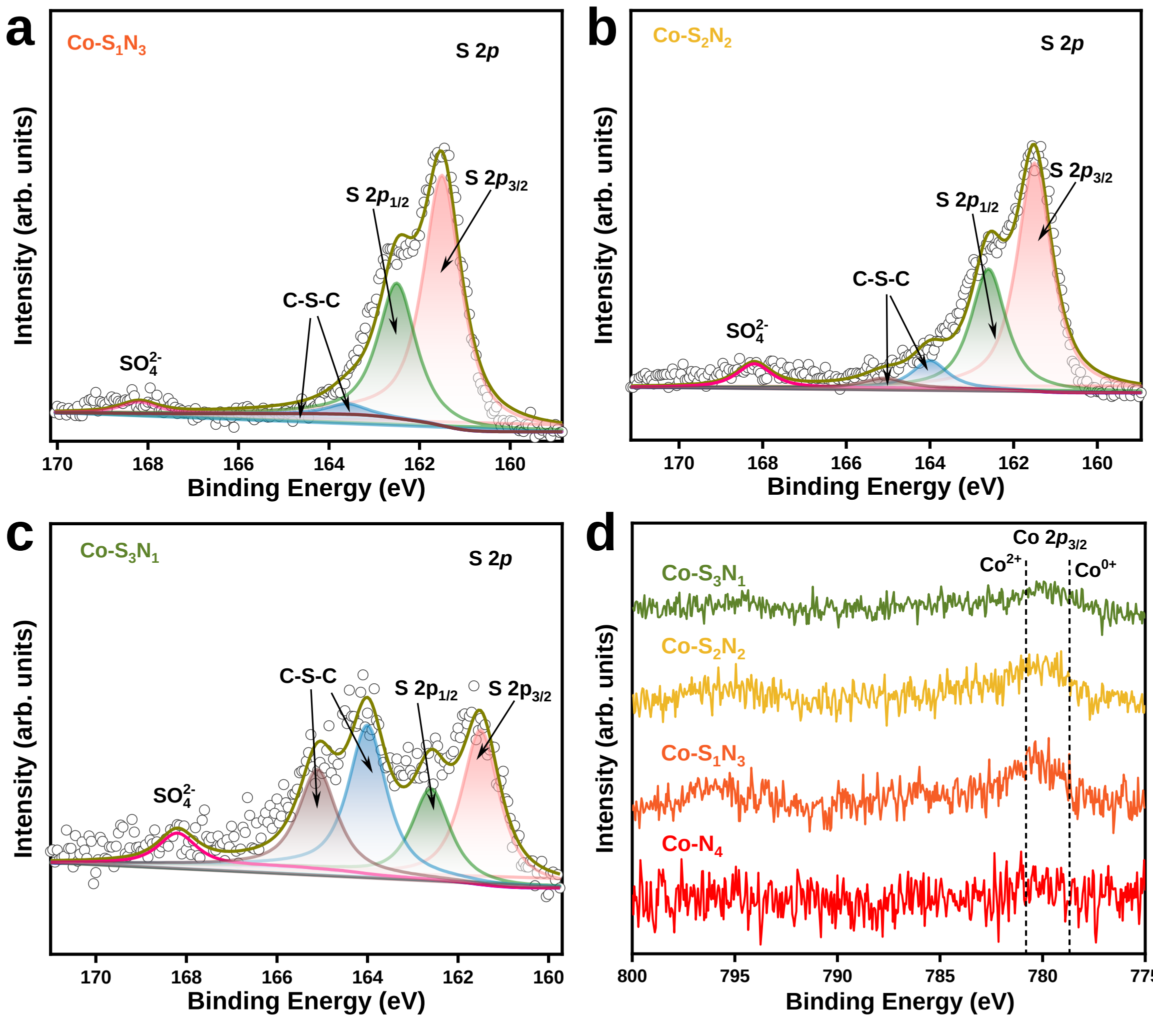

Supplementary Fig. 10. S 2*p* XPS spectra of (a) Co−S_1N_3, (b) Co−S_2N_2, and (c) Co−S_3N_1 SACs. (d) High-resolution Co 2*p* spectra for Co−N_4 Co−S_1N_3, Co−S_2N_2, and Co−S_3N_1.

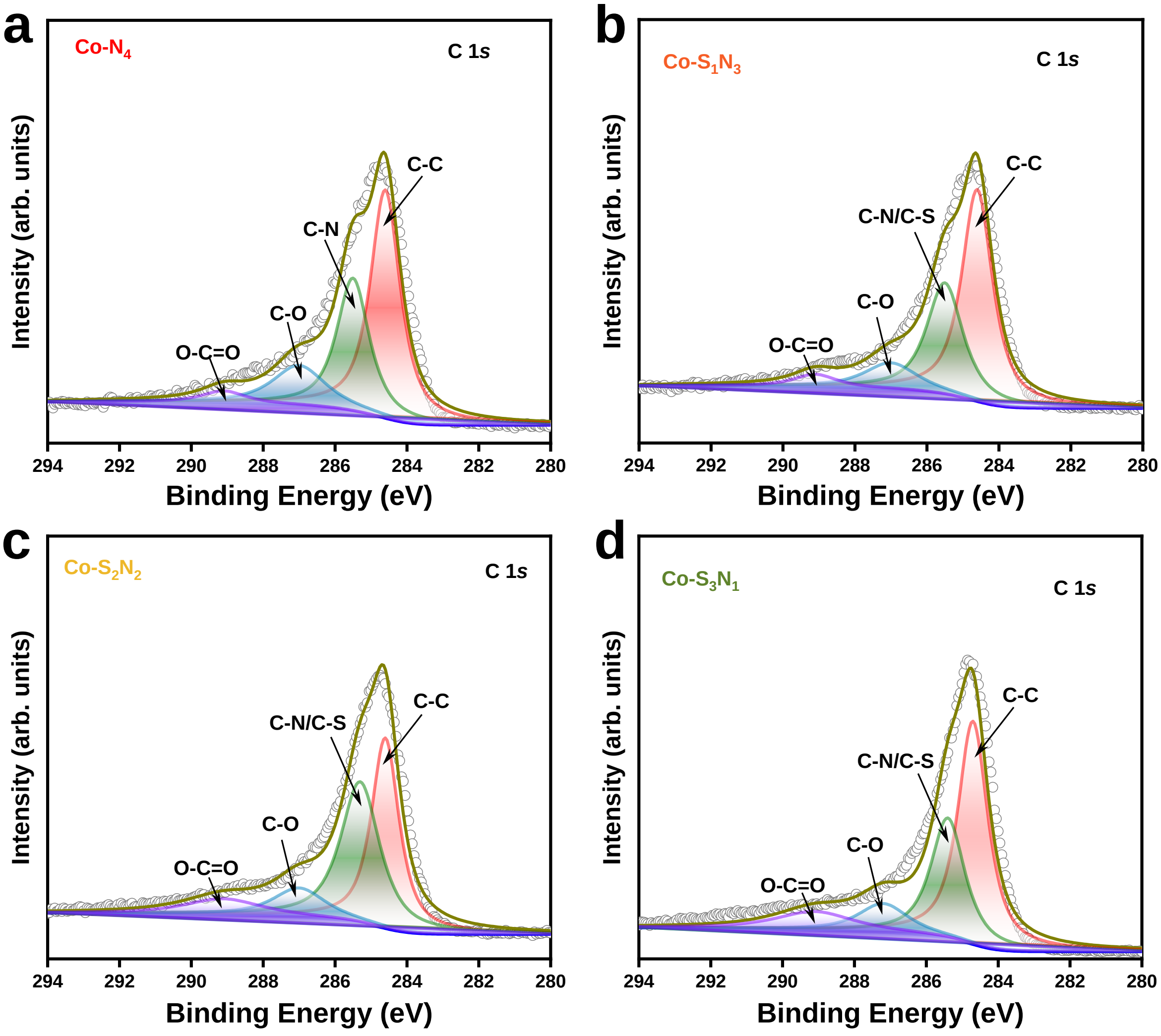

Supplementary Fig. 11. High-resolution C 1*s* XPS spectra of (a) Co−N_4, (b) Co−S_1N_3, (c) Co−S_2N_2, (d) Co−S_3N_1.

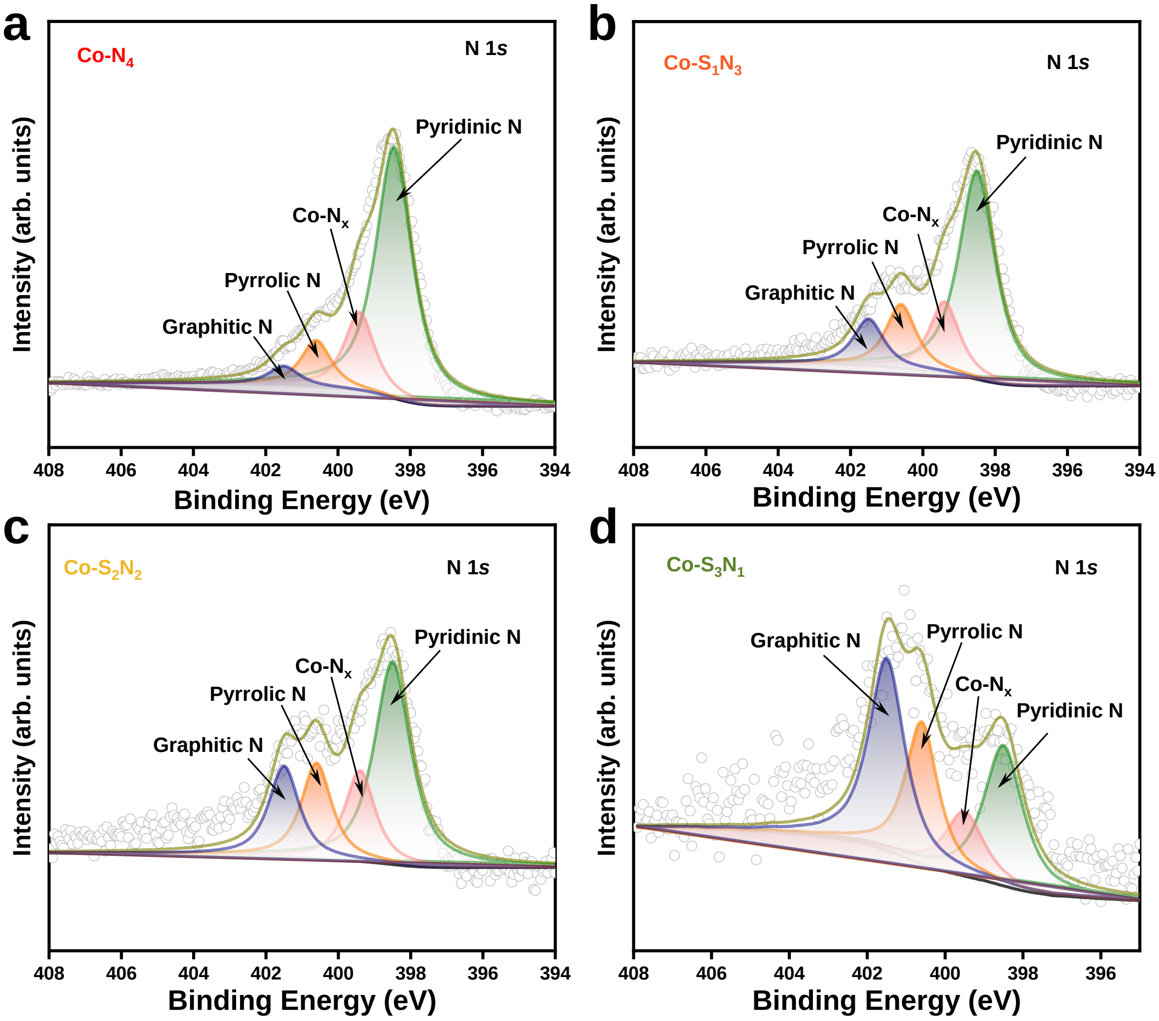

Supplementary Fig. 12. High-resolution N 1*s* XPS spectra of (a) Co−N_4, (b) Co−S_1N_3, (c) Co−S_2N_2, (d) Co−S_3N_1 catalysts.

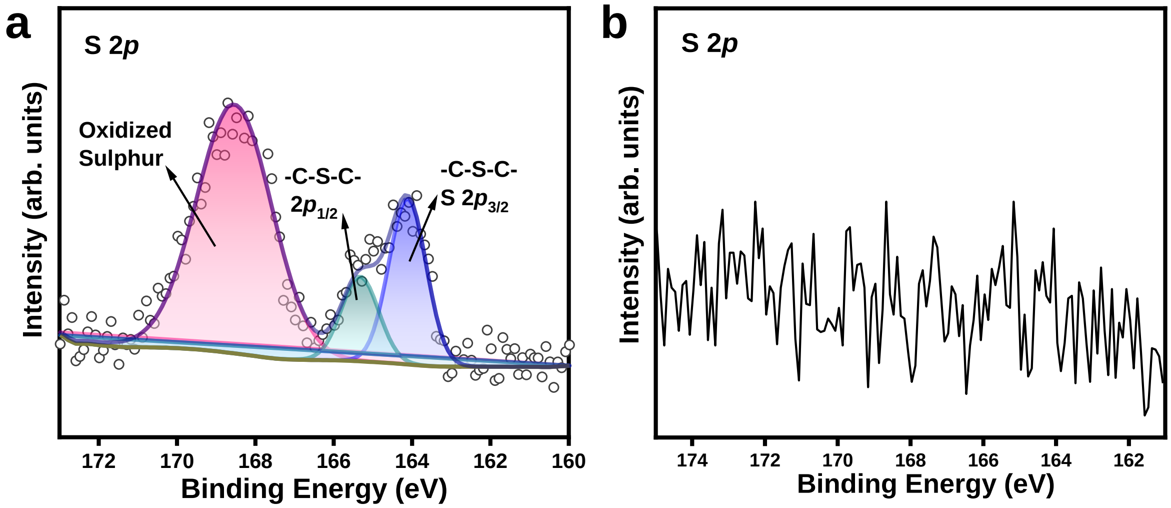

Supplementary Fig. 13. High-resolution S 2*p* XPS spectra of (a) S-doped NC obtained by pyrolysis of ZIF-8 with thiophene; (b) graphite pyrolyzed with thiophene.

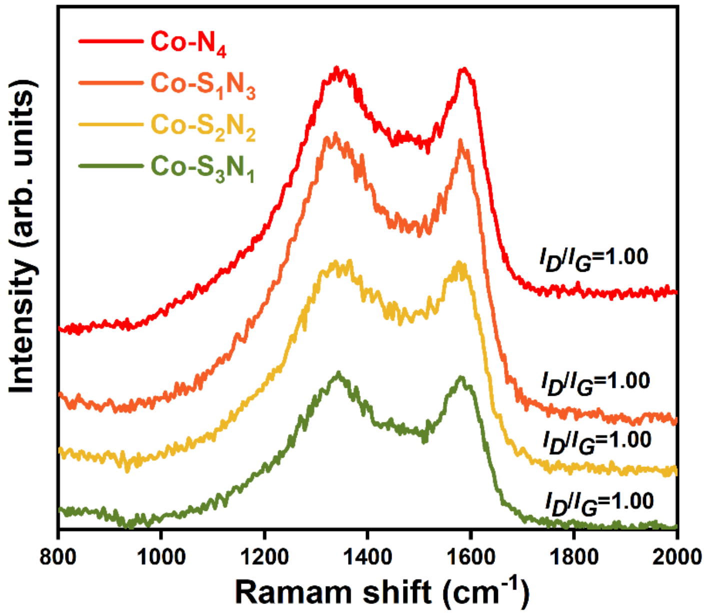

Supplementary Fig. 14. The Raman spectra of $Co{-}N_4$, $Co{-}S_1N_3$, $Co{-}S_2N_2$, and $Co{-}S_3N_1$ SACs.

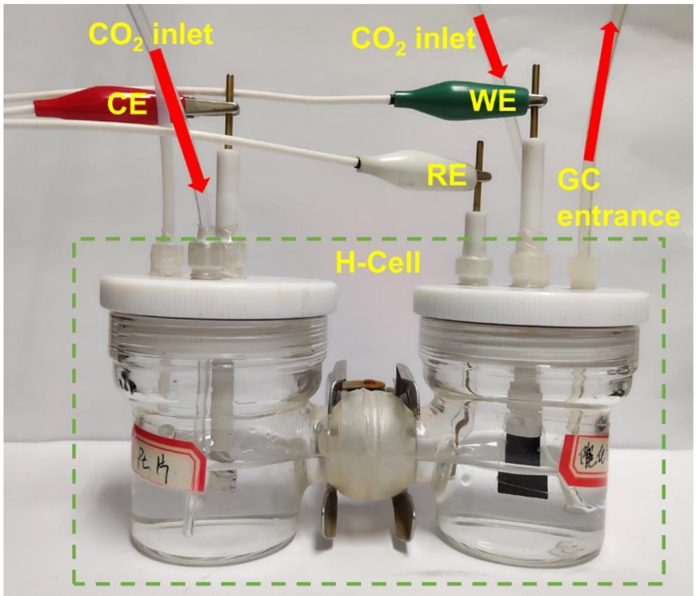

Supplementary Fig. 15. Photograph of the setup for the electrochemical CO_2RR measurements using H-cell with typical three-electrode system.

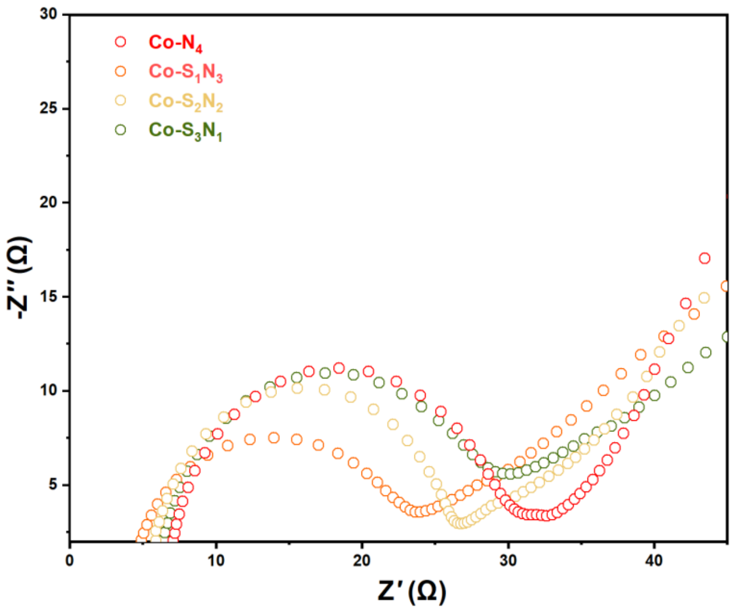

Supplementary Fig. 16. The Nyquist plots of Co−N_4, Co−S_1N_3, Co−S_2N_2, and Co−S_3N_1 SACs at −0.5 V vs RHE.

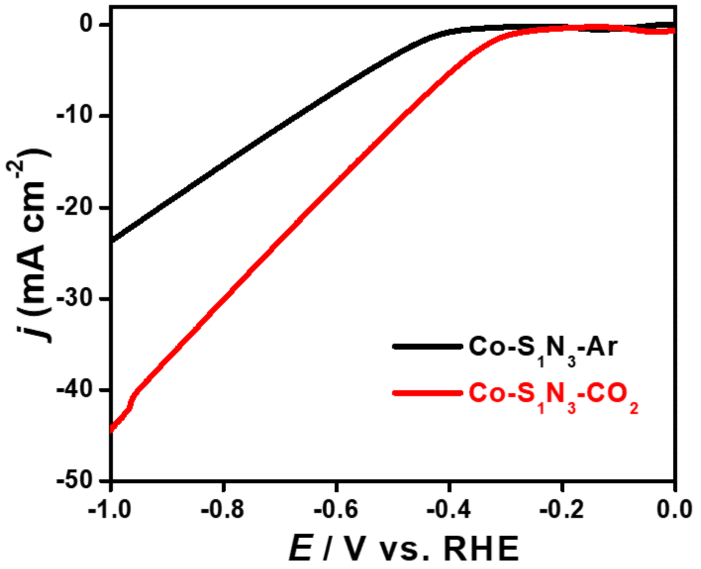

Supplementary Fig. 17. LSV curves of Co−S_1N_3 tested in Ar- and CO_2-saturated 0.5 M $KHCO_3$, respectively.

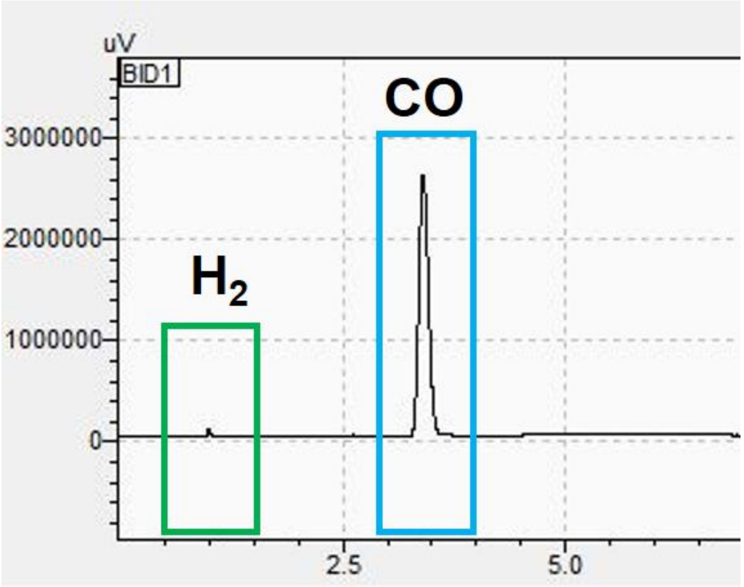

Supplementary Fig. 18. Typical on-line GC result at the potential of −0.52 V using Co−S_1N_3 SAC. The area of CO (at 3.2 min) is much greater than that of H_2 (at 0.9 min).

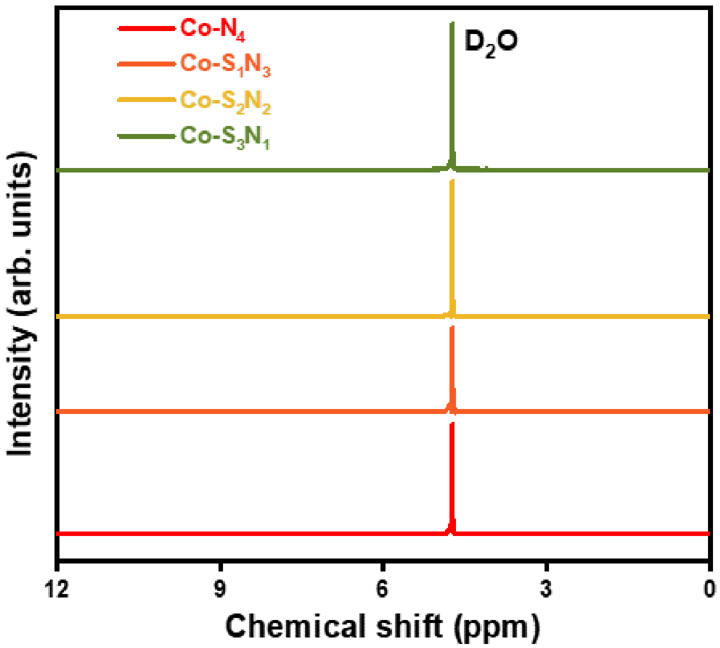

Supplementary Fig. 19. The ^{1}H NMR spectra of the electrolyte after CO_2RR electrolysis by using Co−N_4, Co−S_1N_3, Co−S_2N_2 and Co−S_3N_1 SACs. Only the water (H_2O) signal appeared, without other signals assigned to liquid products such as formic acid, ethanol, etc.

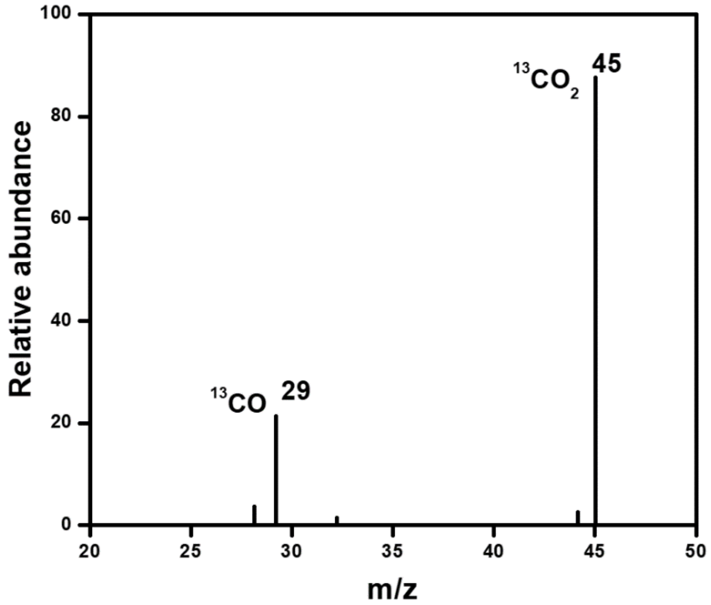

Supplementary Fig. 20. Mass spectra of CO_2RR product using $^{13}CO_2$ as the electrocatalytic feedstock on Co−S_1N_3 SAC. $^{13}CO_2$ feedstock was adopted to react with KOH solution to form $^{13}CO_2$-saturated $KH^{13}CO_3$ electrolyte first. $^{13}CO_2$ was continuously bubbled during the CO_2RR test. The peaks at 29 and 45 were assigned to ^{13}CO product and $^{13}CO_2$ feedstock, respectively. A small amount of air (N_2 at 28, O_2 at 32 and $^{12}CO_2$ at 44 position) was detected due to the cell was not well air tight.

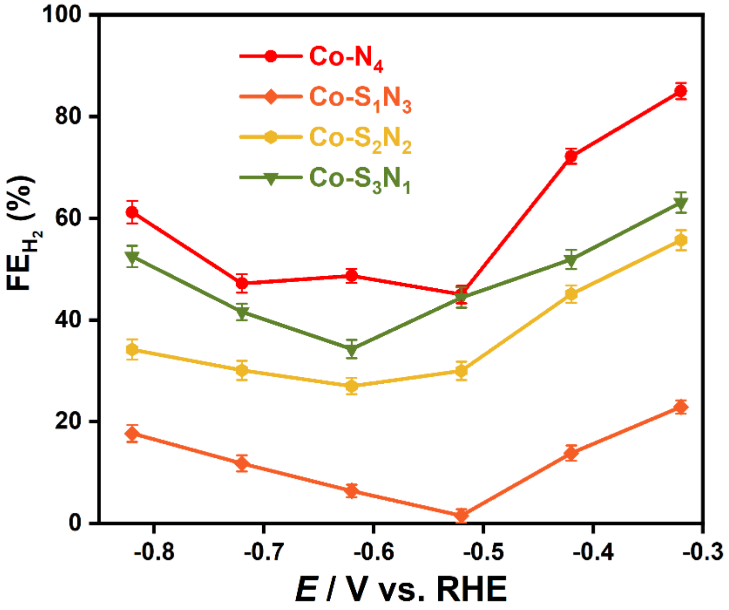

Supplementary Fig. 21. The FE_{H2} of Co–N_4, Co–S_1N_3, Co–S_2N_2, and Co–S_3N_1 SACs measured by GC. Error bars are s.d. of at least three sets of experimental repeats.

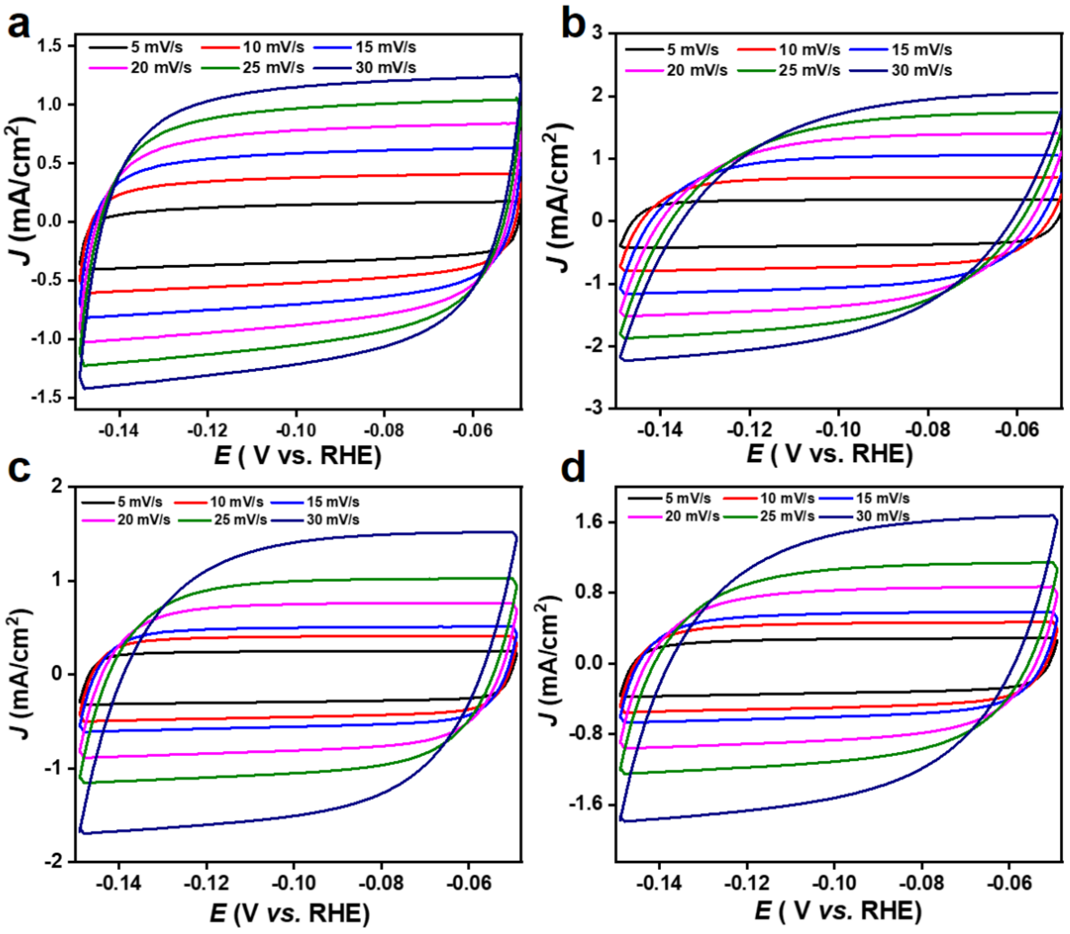

Supplementary Fig. 22. Cyclic voltammograms obtained at different scan rates. (a) Co−N_4, (b) Co−S_1N_3, (c) Co−S_2N_2, (d) Co−S_3N_1.

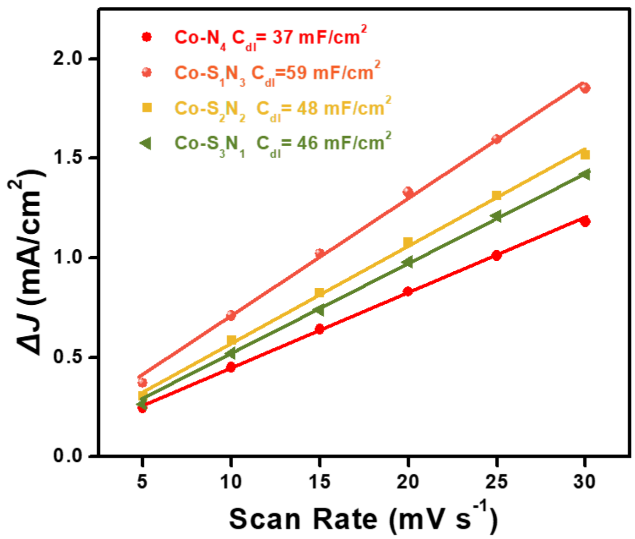

Supplementary Fig. 23 The double-layer capacitance current at −0.1 V plot with the scan rate.

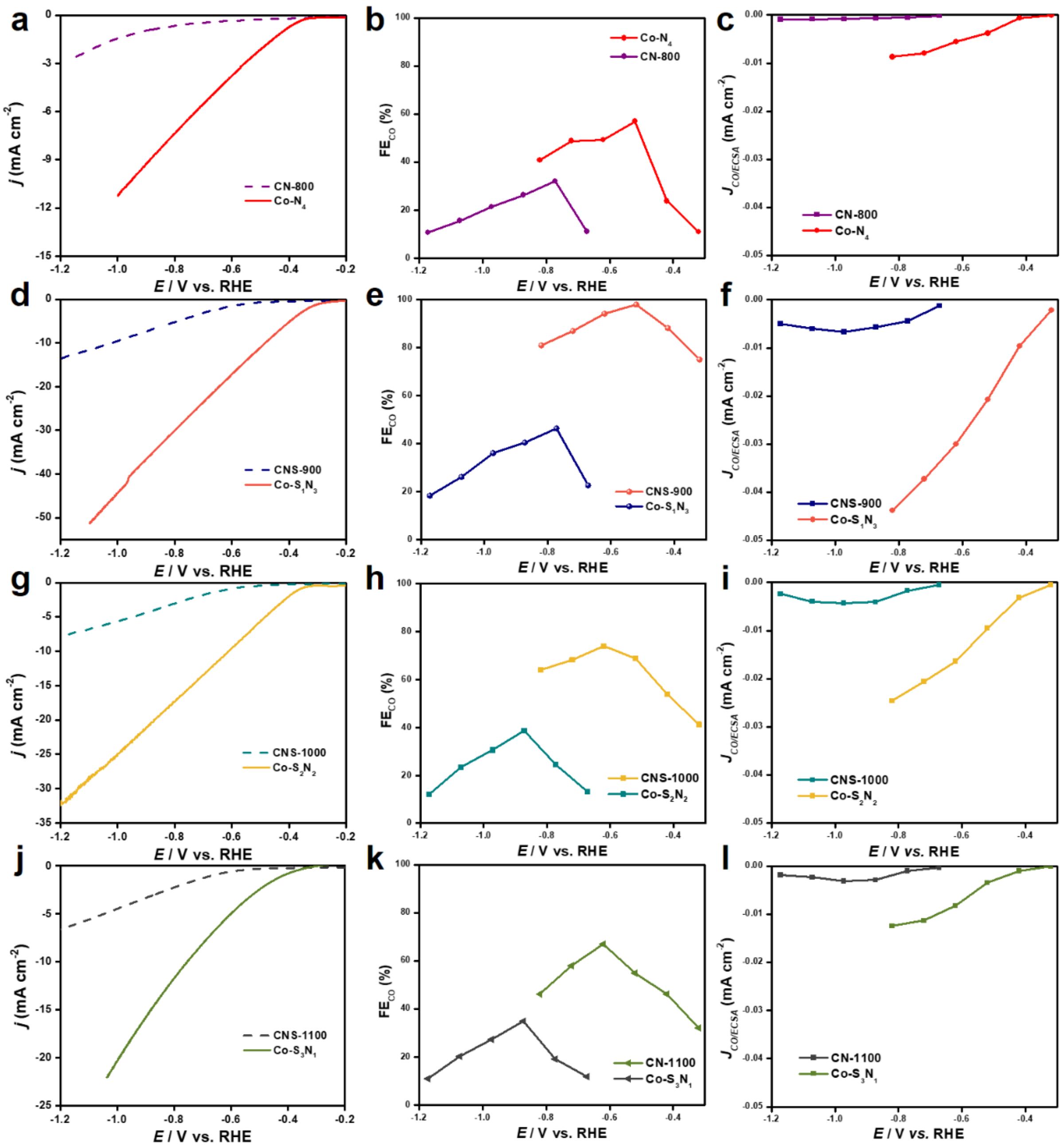

Supplementary Fig. 24. The CO_2RR performances of the Co−S_xN_{4-x} SACs and their N, S doped carbon substrate counterparts. (a, d, g, j) Polarization curves. (b, e, h, k) FE_{CO}. (c, f, i, l) $j_{CO/ECSA}$.

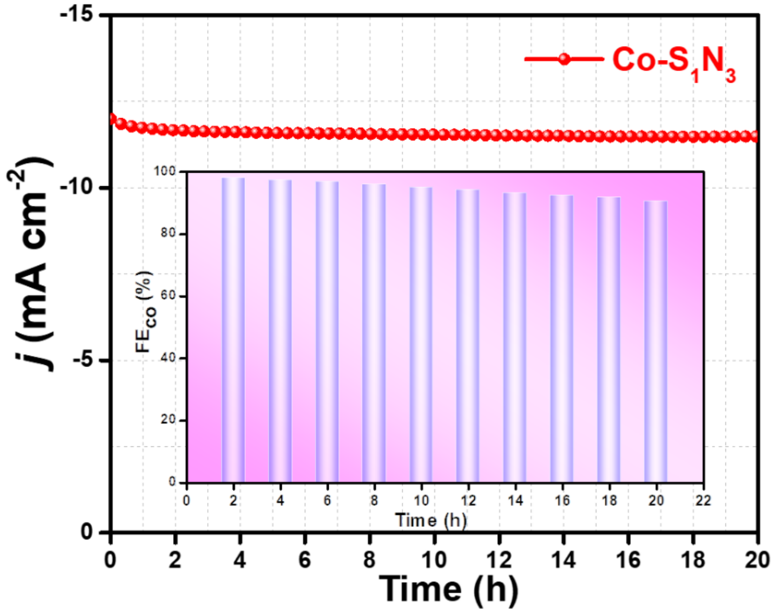

Supplementary Fig. 25. Stability measurement at −0.52 V of Co−S_1N_3 SAC for 20 h. The inset image is corresponding FE_{CO}, which measured ever 2 h of electrolysis.

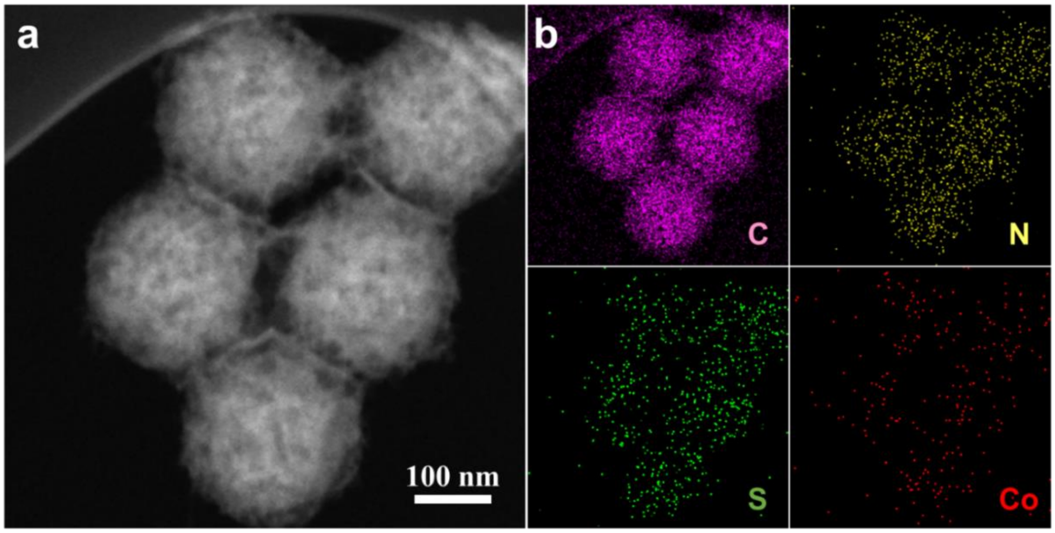

Supplementary Fig. 26. EDS mappings of Co−S_1N_3 SAC after CO_2RR stability test.

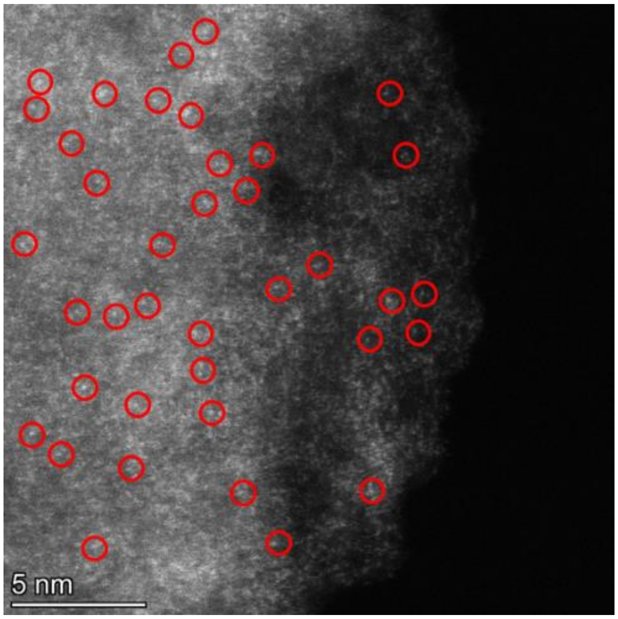

Supplementary Fig. 27. AC HAADF-STEM image of the Co–S_1N_3 SAC after CO_2RR stability test.

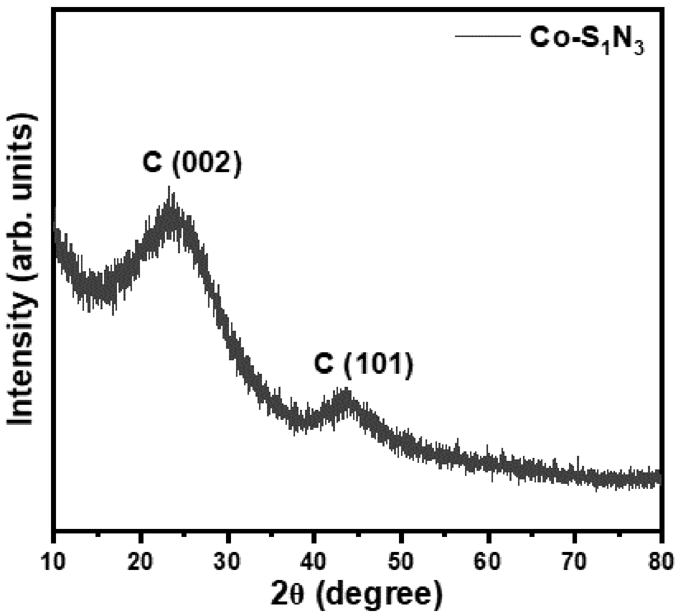

Supplementary Fig. 28. XRD pattern of the Co−S_1N_3 SAC after CO_2RR stability test.

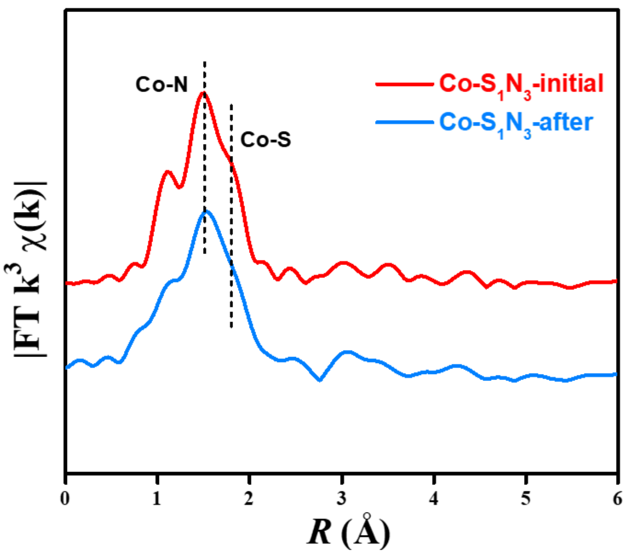

Supplementary Fig. 29. The EXAFS curves of the Co−S_1N_3 SAC after CO_2RR stability test.

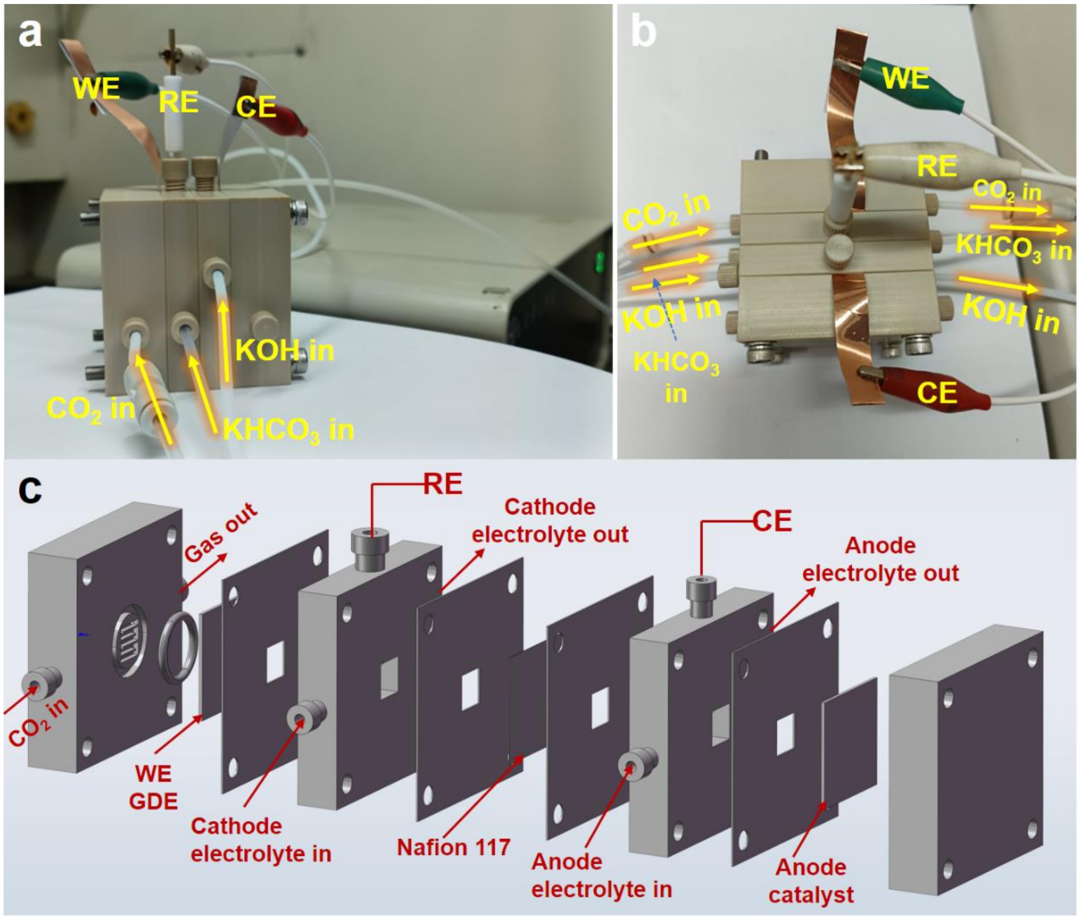

Supplementary Fig. 30. The flow cell setup. (a) side view. (b) top view. (c) scheme shown the detailed structure.

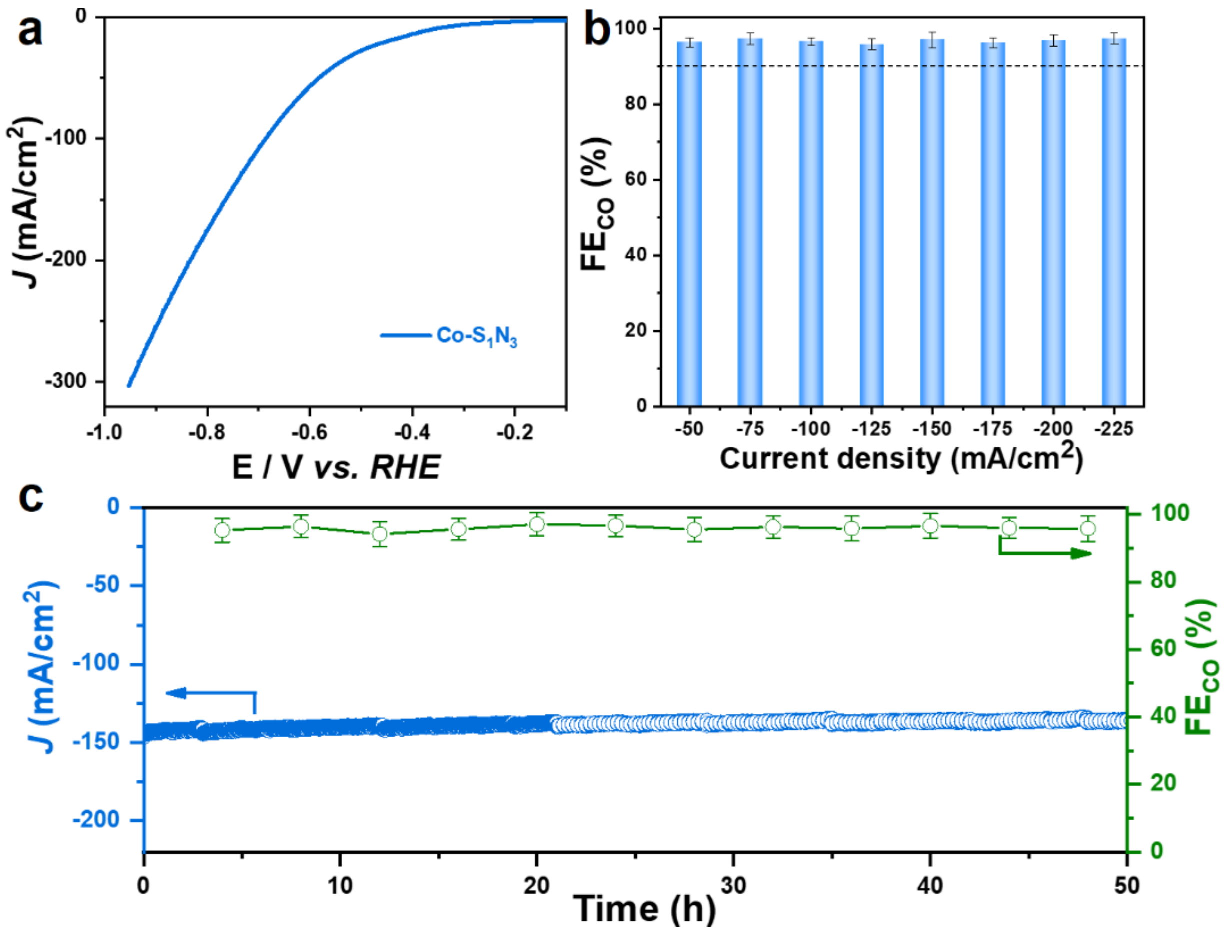

Supplementary Fig. 31. CO_2RR performance of Co–S_1N_3 SAC employing flow cell. (a) polarization curve. (b) FE_{CO}. (c) Chronoamperometry at –0.76 V for 50 h. The FE_{CO} was tested ever 4 h of electrolysis.

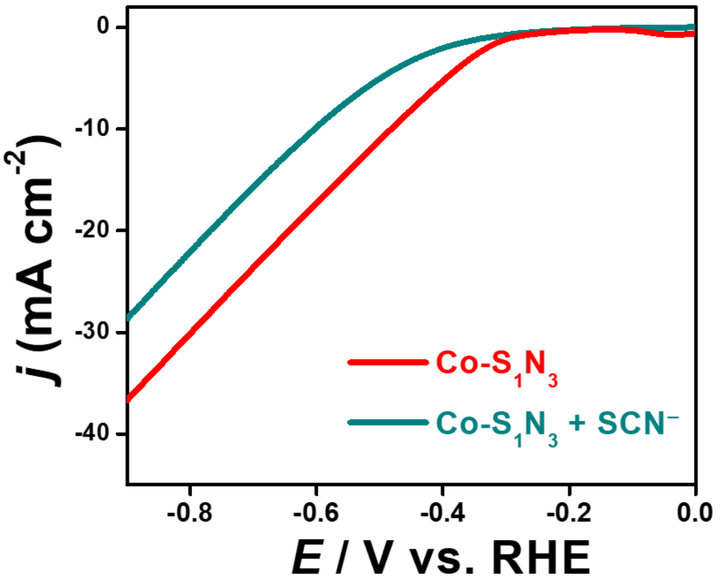

Supplementary Fig. 32. Polarization curves of Co−S_1N_3 measured in CO_2-saturated 0.5 M $KHCO_3$ and 0.5 M $KHCO_3$+ 0.2 M KSCN solution.

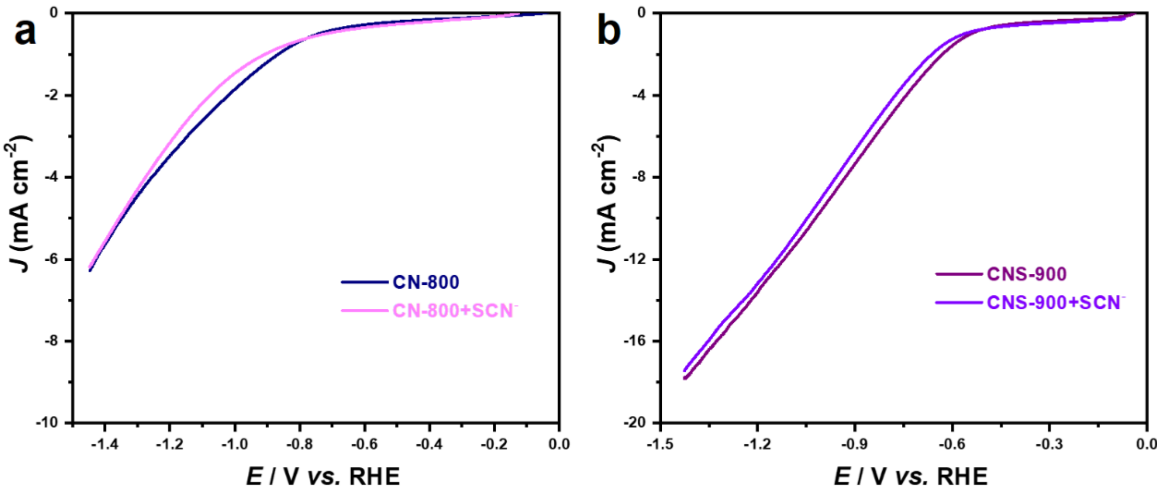

Supplementary Fig. 33. Polarization curves measured in CO_2-saturated 0.5 M $KHCO_3$ and 0.5 M $KHCO_3$ + 0.2 M KSCN solution. (a) CN-800. (b) CNS-900.

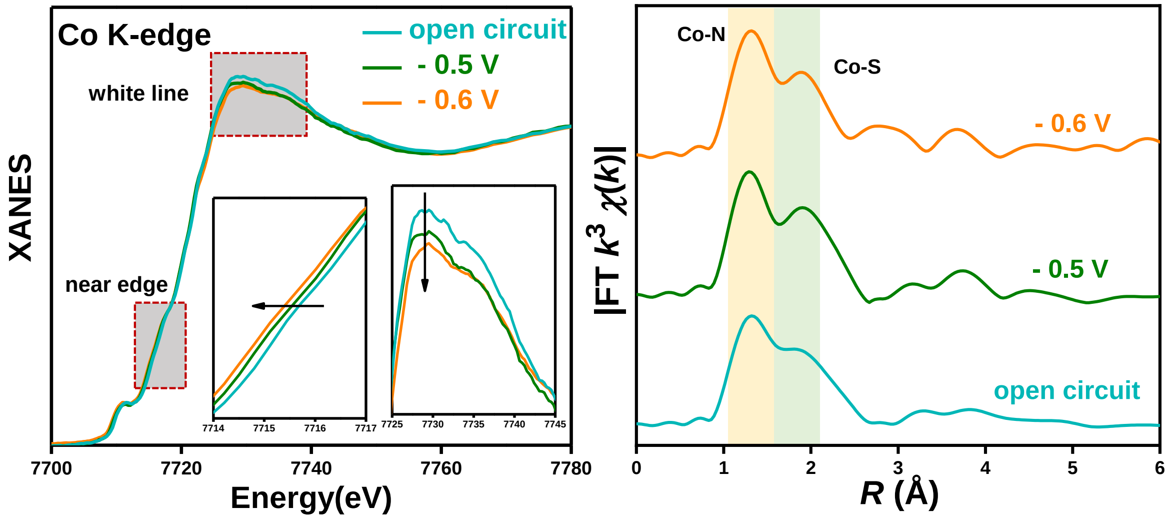

Supplementary Fig. 34. (a) *In-situ* Co K-edge XANES of the Co−S_1N_3 SAC under different potentials. The inset is the magnified near edge and white line regions. (b) FT-EXAFS spectra of the Co−S_1N_3 SAC under different potentials.

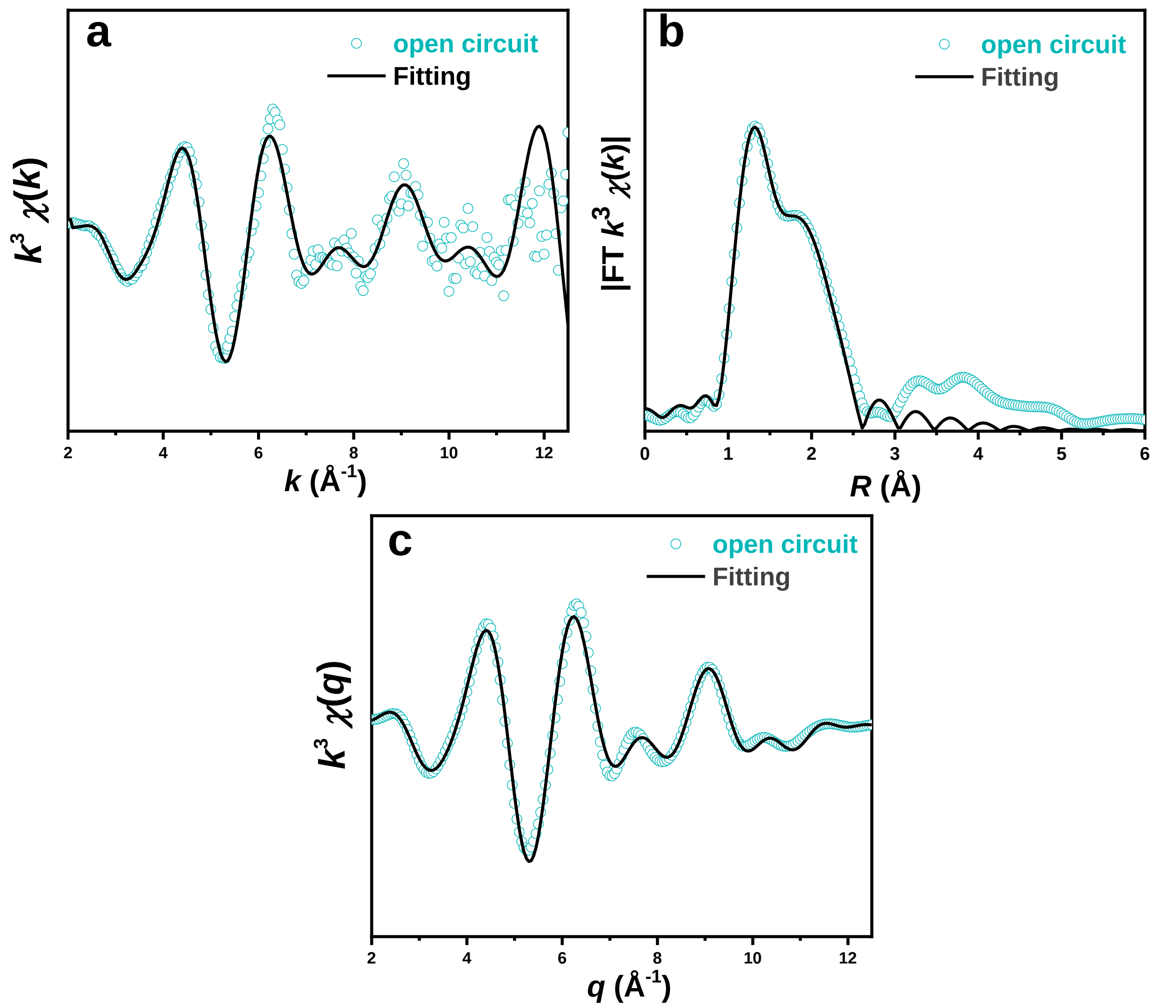

Supplementary Fig. 35. In-situ FT-EXAFS fitting spectra of Co−S_1N_3 under open circuit. (a) *k* space, (b) *R* space, (c) *q* space.

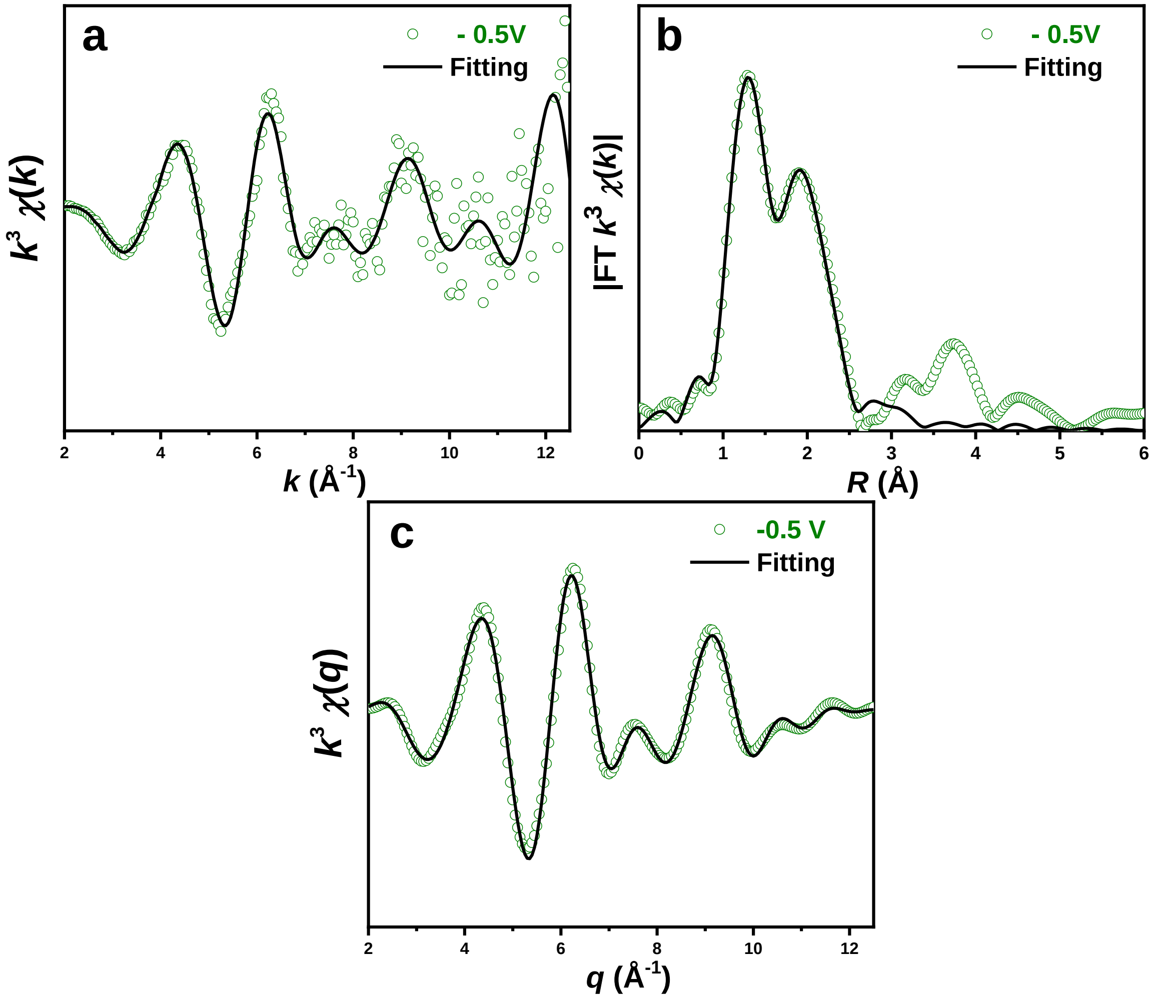

Supplementary Fig. 36. In-situ FT-EXAFS fitting spectra of Co−S_1N_3 at –0.5 V (a) *k* space, (b) *R* space, (c) *q* space.

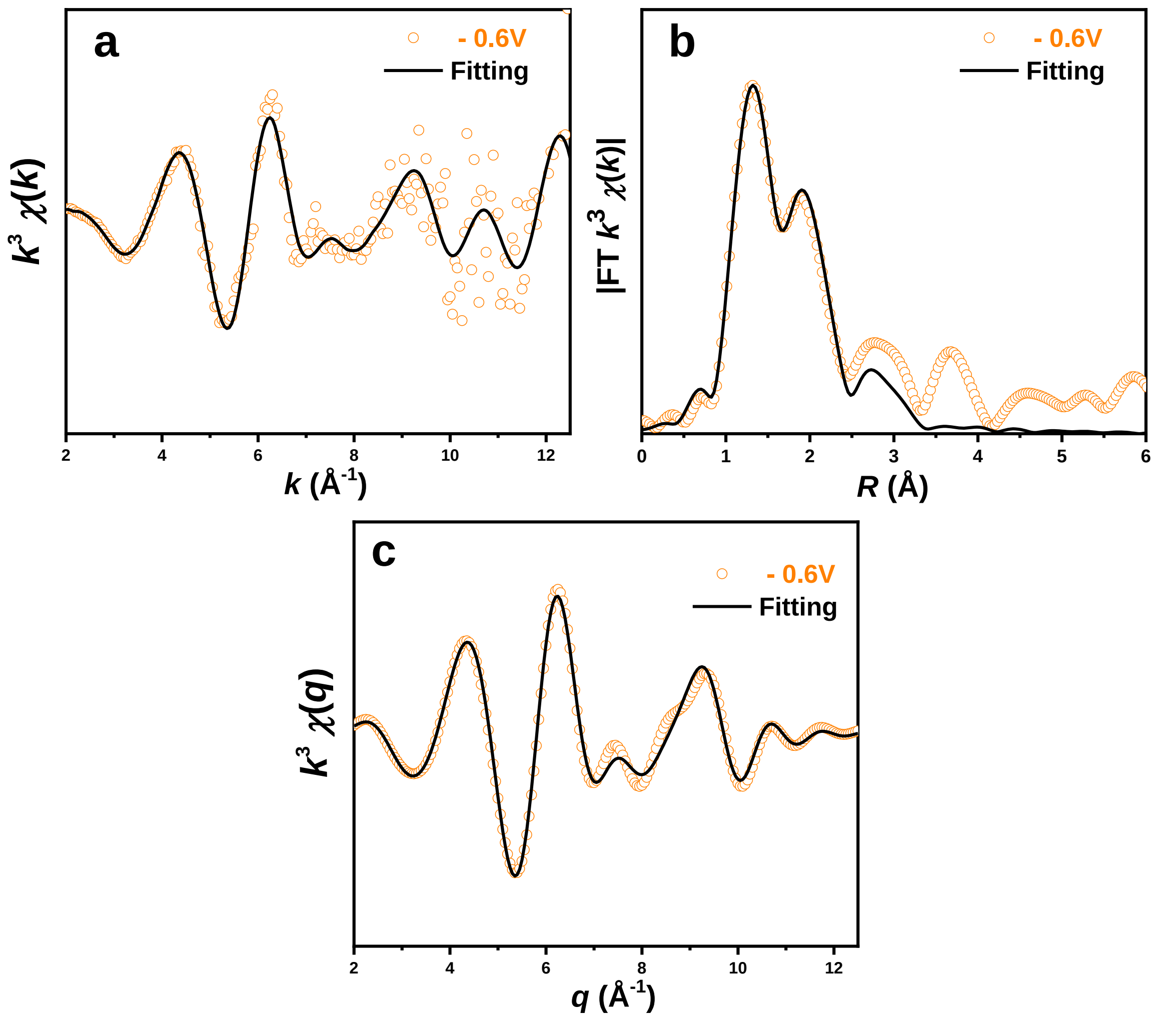

Supplementary Fig. 37. In-situ FT-EXAFS fitting spectra of Co−S_1N_3 at –0.6 V (a) *k* space, (b) *R* space, (c) *q* space.

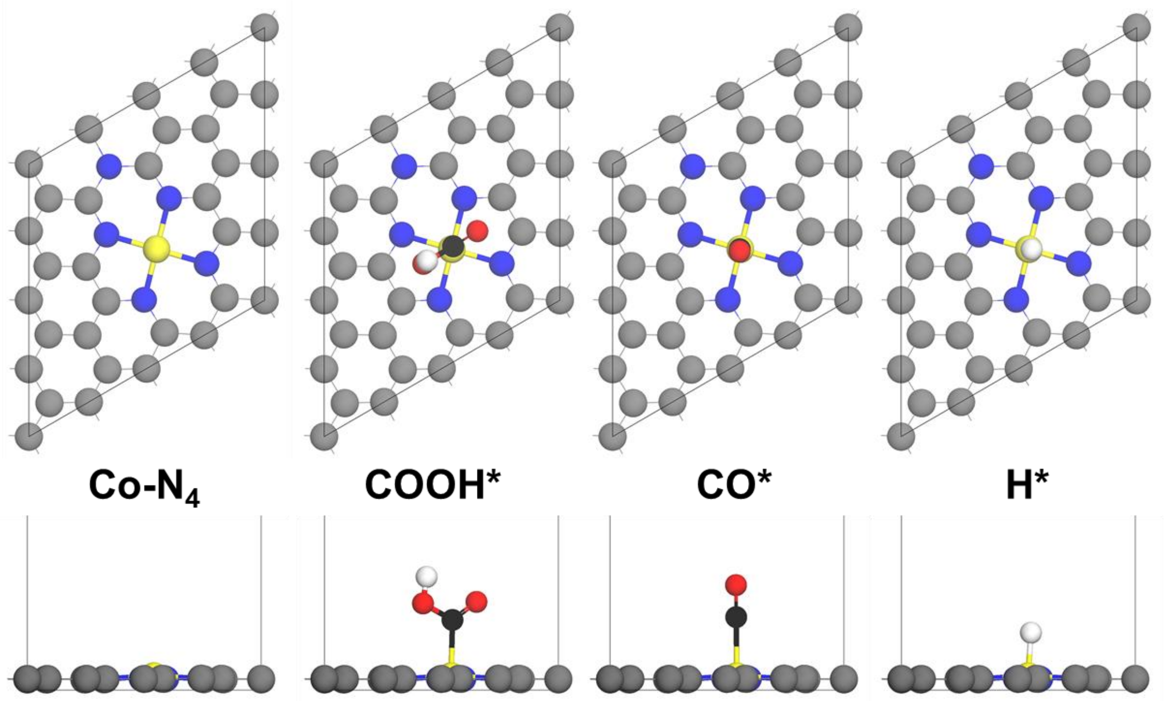

Supplementary Fig. 38. Adsorption configurations of COOH, CO, and H on Co−N_4 (Yellow for Co, gray for C in NC, blue for N, white for H, red for O, and black for C in reaction intermediates).

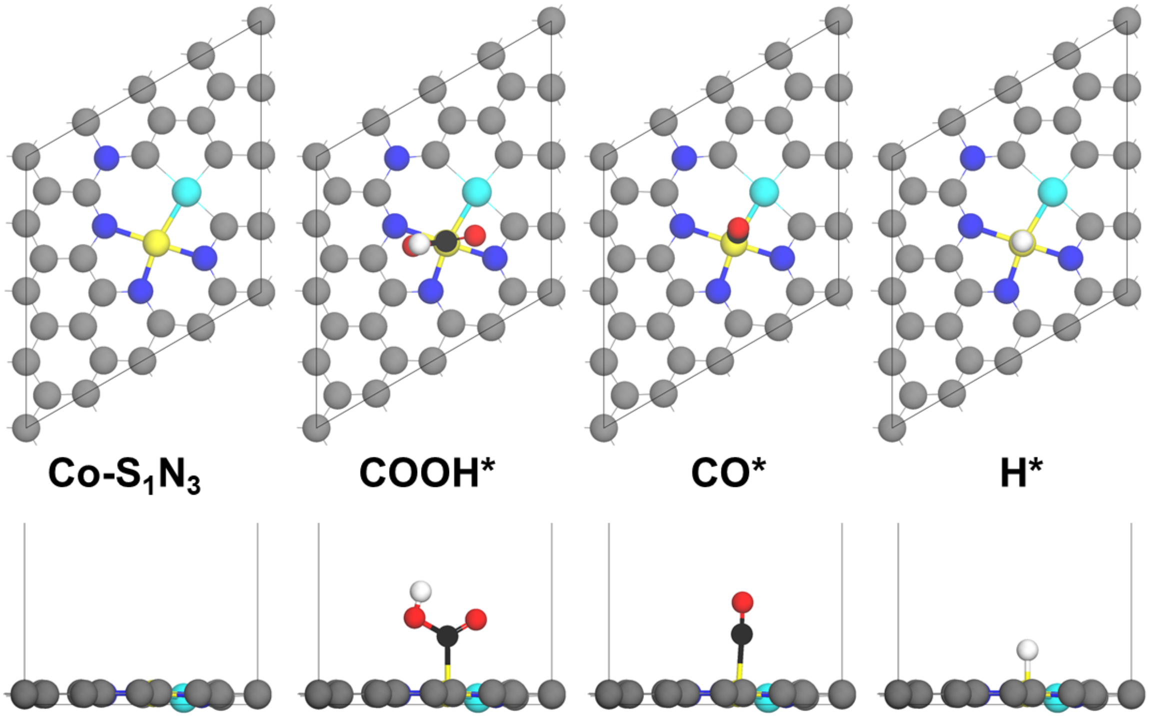

Supplementary Fig. 39. Adsorption configurations of COOH, CO, and H on Co−S_1N_3 (Yellow for Co, Cyan for S, gray for C in NC, blue for N, white for H, red for O, and black for C in reaction intermediates).

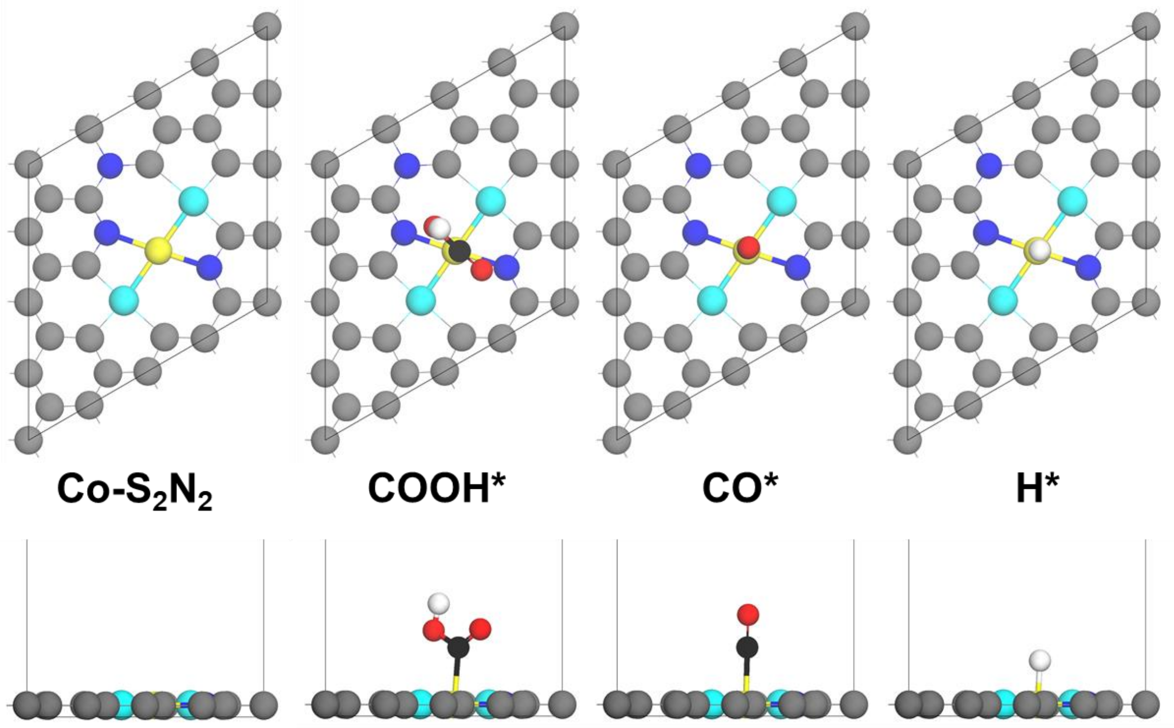

Supplementary Fig. 40. Adsorption configurations of COOH, CO, and H on Co−S_2N_2 (Yellow for Co, Cyan for S, gray for C in NC, blue for N, white for H, red for O, and black for C in reaction intermediates).

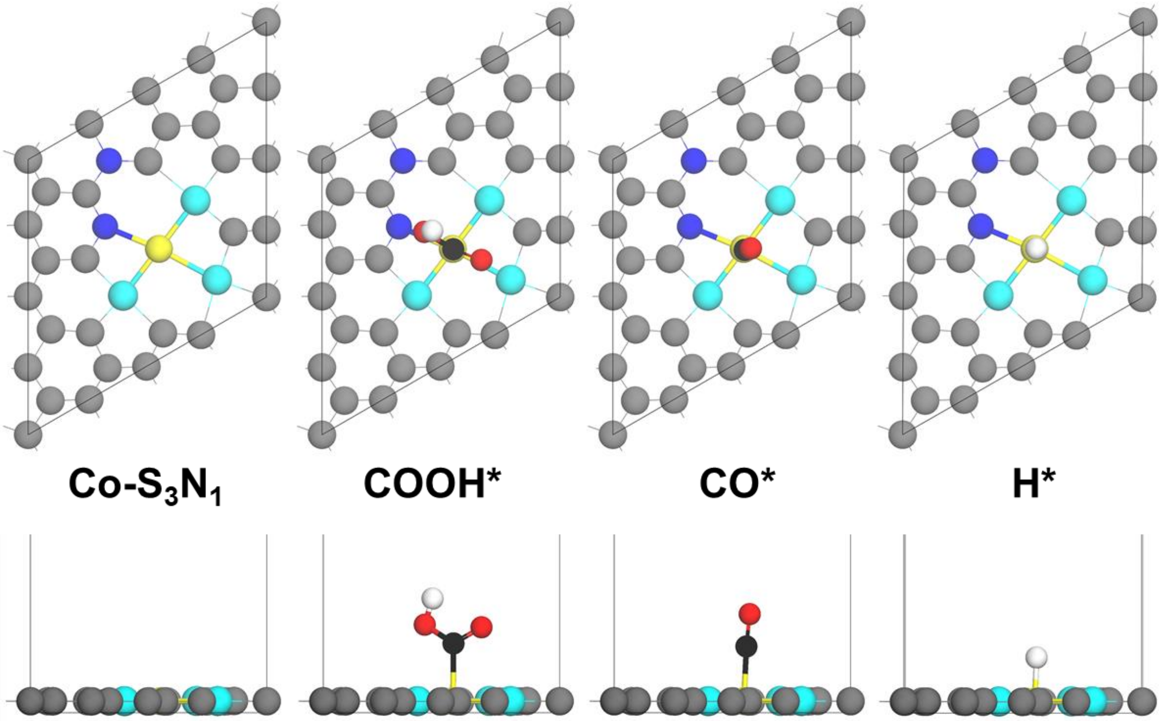

Supplementary Fig. 41. Adsorption configurations of COOH, CO, and H on Co−S_3N_1 (Yellow for Co, Cyan for S, gray for C in NC, blue for N, white for H, red for O, and black for C in reaction intermediates).

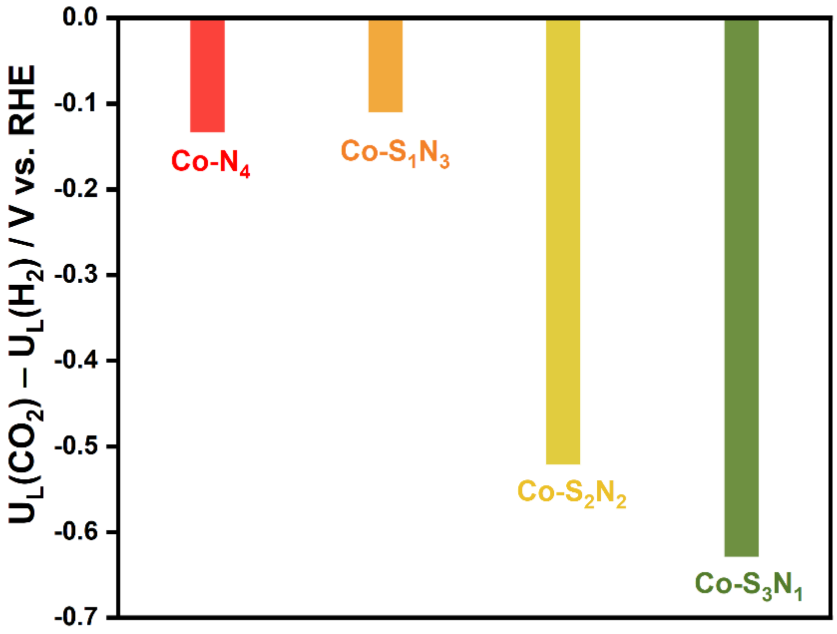

Supplementary Fig. 42. Limiting potentials difference for CO_2 reduction and HER on Co−S_xN_{4-x} SACs.

Supplementary Table 1 The micro/mesoporous volume, S_{BET}, S_{micro}, S_{ext} for the Co−S_xN_{4-x} SACs

**Samples**	**Microporous volume (cm^3 g^{-1})**	**Mesoporous volume (cm^3 g^{-1})**	**S_{BET} (m^2 g^{-1})**	**S_{micro} (m^2 g^{-1})**	**S_{ext} (m^2 g^{-1})**
Co−N_4	0.44	0.08	862	635	225
Co−S_1N_3	0.29	0.26	919	624	295
Co−S_2N_2	0.25	0.33	928	601	327
Co−S_3N_1	0.16	1.48	988	526	462

Supplementary Table 2. The weight content of Co in $Co-N_4$, $Co-S_1N_3$, $Co-S_2N_2$ and $Co-S_3N_1$ measured by ICP-OES analysis.

**Sample**	**$Co-N_4$**	**$Co-S_1N_3$**	**$Co-S_2N_2$**	**$Co-S_3N_1$**
Co content (wt %)	0.56	0.54	0.53	0.54

Supplementary Table 3 Light elements contents in the Co−S_xN_{4-x} SACs.

Samples	C %	N%	S %	H %
Co−N_4	78.0	17.7	0	0.098
Co−S_1N_3	77.7	11.2	5.0	0.061
Co−S_2N_2	79.0	7.6	5.2	0.031
Co−S_3N_1	80.1	6.0	5.6	0.014

Supplementary Table 4. Structural parameters extracted from the Co K-edge EXAFS fitting. (S_0^2=0.85).

Sample	Scattering pair	CN	*R*(Å)	σ^2(10^{-3}Å^2)	ΔE_0(eV)	R factor
Co−S$_1$N$_3$	Co−N	3.0±0.6	1.98±0.02	5.3±0.7	1.0±0.2	0.009
	Co−O	1.0±0.2	2.08±0.02	5.9±0.7		
	Co−S	1.1±0.2	2.33±0.02	5.6±0.8		
Co−S$_2$N$_2$	Co−N	2.1±0.4	1.97±0.02	5.1±0.6	1.5±0.3	0.008
	Co−O	1.1±0.2	2.09±0.02	6.3±0.9		
	Co−S	2.0±0.4	2.30±0.02	5.3±0.9		
Co−S$_3$N$_1$	Co−N	1.1±0.2	1.99±0.02	5.4±0.6	1.0±0.2	0.007
	Co−O	1.0±0.2	2.08±0.02	5.9±0.7		
	Co−S	3.1±0.6	2.31±0.02	5.8±0.7		
Co−N$_4$	Co−N	4.1±0.7	1.98±0.02	5.3±0.6	0.5±0.1	0.006
	Co−O	1.0±0.2	2.07±0.02	5.5±0.7		
Co foil	Co−Co	12*	2.49±0.02	4.9±0.5	1.0±0.2	0.004

S_0^2 is the amplitude reduction factor; CN is the coordination number; *R* is interatomic distance (the bond length between Co central atoms and surrounding coordination atoms); σ^2 is Debye-Waller factor (a measure of thermal and static disorder in absorber-scatterer distances); ΔE_0 is edge-energy shift (the difference between the zero kinetic energy value of the sample and that of the theoretical model). R factor is used to value the goodness of the fitting.

* This value was fixed during EXAFS fitting, based on the known structure of Co foil.

Error bounds that characterize the structural parameters obtained by EXAFS spectroscopy were estimated as $N \pm 20\%$; $R \pm 1\%$; $\sigma^2 \pm 20\%$; $\Delta E_0 \pm 20\%$.

Supplementary Table 5. The N specie content from XPS.

Samples	Pyridinic N (%)	Co−N_x (%)	Pyrrolic N (%)	Graphitic N (%)
Co−N_4	67.8	17.6	10.4	4.2
Co−S_1N_3	57.9	16.5	14.8	10.8
Co−S_2N_2	47.8	15.6	20.5	16.1
Co−S_3N_1	28.1	13.0	23.7	35.2

Supplementary Table 6 The ECSA normalized CO partial current density at −0.72 V of the Co−S_xN_{4-x} SACs and their N, S doped carbon substrate counterparts.

	**$-j_{CO/ECSA}$ (mA cm^{-2})**		
	**Co−S_xN_{4-x} SACs (j_{SAC})**	**N, S doped carbon substrate counterparts (j_{sub})**	j_{sub}/j_{SAC}
Co-N_4	7.9×10^{-3}	5.4×10^{-4}	6.8%
Co-S_1N_3	3.7×10^{-2}	2.8×10^{-3}	7.6%
Co-S_2N_2	2.1×10^{-2}	1.1×10^{-3}	5.2%
Co-S_3N_1	1.1×10^{-2}	6.5×10^{-4}	5.9%

Supplementary Table 7. Summary of the catalytic performance of the reported CO_2RR catalysts.

**Catalysts**	**Electrolyte**	**Potential (V)**	**Overpotential (mV)**	**FE_{CO} (%)**	**TOF (h^{-1})**	**Ref**
Co−S_1N_3	0.5 M $KHCO_3$	−0.52	410	98	4564	This work
		−0.72	610	87	8505	
CoPP@CNT	0.5 M $NaHCO_3$	−0.6	490	98.3	4932	*Angew. Chem. Int. Ed.* **2019**, *58*, 6595.
CoPPc	0.5 M $KHCO_3$	−0.61	500	~90	5040	*Chem* **2017**, *3*, 652.
CoPc-F	0.5 M $KHCO_3$	−0.90	790	88	7380	*ACS Catal.* **2016**, *6*, 3092
COF-367-Co	0.5 M $KHCO_3$	−0.67	560	91	1908	*Science*, **2015**, 349, 1208.
CoN_5	0.2 M $NaHCO_3$	−0.73	620	99.0	480.2	*J. Am. Chem. Soc.* **2018**, *140*, 4218.
ZIF-NC-Fe-Co	0.1 M $KHCO_3$	−0.70	590	76	200	*Angew. Chem. Int. Ed.* **2022**, *61, e202205632*
$Al_2(OH)_2$TCPP-Co	0.1 M $KHCO_3$	−0.71	600	76	200	*J. Am. Chem. Soc.* **2015**, *137*, 14129.
NapCo@SNG	0.1 M $KHCO_3$	−0.735	625	95	1620	*Angew. Chem. Int. Ed.* **2019**, *58*, 13532.
A-Ni-NSG	0.5 M $KHCO_3$	−0.72	610	94	2960	*Nat. Energy*, **2018**, 3, 140.
Ni SAs/N-C	0.5 M $KHCO_3$	−1.00	890	71.9	5273	*J. Am. Chem. Soc.* **2017**, *139*, 8078.
Fe^{3+}-N-C	0.5 M $KHCO_3$	−0.45	340	90.0	~1030	*Science*, **2019**, *364*, 1091.
Ni/Fe-N-C	0.5 M $KHCO_3$	−0.70	590	98	2900	*Angew. Chem. Int. Ed.* **2019**, *58*, 6972
Tri-Ag-NPs	0.1 M $KHCO_3$	−0.856	746	96.8	N/A	*J. Am. Chem. Soc.* **2017**, *139*, 2160.

Supplementary Table 8. Structural parameters extracted from the Co K-edge EXAFS fitting at different conditions during CO_2RR process. (S_0^2=0.85).

Sample	Scattering pair	CN	*R*(Å)	σ^2(10^{-3}Å^2)	ΔE_0(eV)	R factor
Co−S_1N_3 at −0.6 V	Co−N	2.9±0.6	1.98±0.02	5.9±0.8	1.5±0.2	0.009
	Co−C	1.1±0.2	2.08±0.02	6.9±0.9		
	Co−S	1.0±0.2	2.33±0.02	6.2±0.8		
Co−S_1N_3 at −0.5 V	Co−N	3.1±0.6	1.98±0.02	6.4±0.9	1.0±0.2	0.007
	Co−C	0.9±0.2	2.09±0.02	5.3±0.6		
	Co−S	1.1±0.2	2.32±0.02	5.6±1.0		
Co−S_1N_3 at open circuit	Co−N	3.2±0.5	1.99±0.02	5.2±0.7	1.0±0.2	0.006
	Co−C	1.0±0.2	2.08±0.02	5.7±0.8		
	Co−S	1.1±0.2	2.33±0.02	6.5±0.8		

S_0^2 is the amplitude reduction factor; CN is the coordination number; *R* is interatomic distance (the bond length between Co central atoms and surrounding coordination atoms); σ^2 is Debye-Waller factor (a measure of thermal and static disorder in absorber-scatterer distances); ΔE_0 is edge-energy shift (the difference between the zero kinetic energy value of the sample and that of the theoretical model). *R* factor is used to value the goodness of the fitting.

Error bounds that characterize the structural parameters obtained by EXAFS spectroscopy were estimated as $N \pm 20\%$; $R \pm 1\%$; $\sigma^2 \pm 20\%$; $\Delta E_0 \pm 20\%$.

Supplementary Notes

Supplementary Note 1 | XAFS fitting parameters

The fitting ranges for Supplementary Table 4:

Co–S_1N_3 (k-space range: 2.0-12.5 Å^{-1}; R-space range: 0.5-2.5 Å), N_{idp}=13;

Co–S_2N_2 (k-space range: 2.0-12.5 Å^{-1}; R-space range: 0.5-2.5 Å), N_{idp}=13;

Co–S_3N_1 (k-space range: 2.0-12.5 Å^{-1}; R-space range: 0.5-2.5 Å), N_{idp}=13;

Co–N_4 (k-space range: 2.0-12.5 Å^{-1}; R-space range: 0.5-2.5 Å), N_{idp}=10;

Co foil (k-space range: 2.0-14.2 Å^{-1}; R-space range: 1.0-3.0 Å), N_{idp}=15;

The fitting ranges for Supplementary Table 8:

Co–S_1N_3 at –0.6 V vs. RHE (k-space range: 2.0-12.5 Å^{-1}; R-space range: 0.2-2.5 Å), N_{idp}=13;

Co–S_1N_3 at –0.5 V vs. RHE (k-space range: 2.0-12.5 Å^{-1}; R-space range: 0.2-2.5Å), N_{idp}=13;

Co–S_1N_3 at open circuit (k-space range: 2.0-12.5 Å^{-1}; R-space range: 0.2-2.5 Å), N_{idp}=13;

The number of independent points (N_{idp}) is given by

$$N_{\mathrm{idp}} = \frac{2\Delta R\Delta k}{\pi}$$

References:

(1) Rehr, J. J. & Albers, R. C. Theoretical approaches to X-ray absorption fine structure. *Rev. Mod. Phys.* **72**, 621-654 (2000).

(2) Joly, Y. X-ray absorption near-edge structure calculations beyond the muffin-tin approximation. *Phys. Rev. B*, **63**, 125120 (2001).

(3) Bunău, O. & Joly, Y. Self-consistent aspects of X-ray absorption calculations. *J. Phys. Condens. Matter.* **21**, 345501 (2009).
